# Endothelin-A Receptor Antagonist Alleviates Allergic Airway Inflammation *via* the Inhibition of ILC2 Function

**DOI:** 10.3389/fimmu.2022.835953

**Published:** 2022-02-11

**Authors:** Xiaogang Zhang, Ziyang Chen, Shaowen Zuo, Hengbiao Sun, Xinyao Li, Xiao Lu, Zhe Xing, Meiqi Chen, Jingping Liu, Gang Xiao, Yumei He

**Affiliations:** ^1^ Department of Immunology, School of Basic Medical Sciences, Southern Medical University, Guangzhou, China; ^2^ Department of Neurosurgery Affiliated Dongguan Hospital, Southern Medical University, Dongguan, China; ^3^ Department of Clinical Laboratory, The Third Affiliated Hospital of Southern Medical University, Southern Medical University, Guangzhou, China; ^4^ Guangdong Provincial Key Laboratory of Single Cell Technology and Application, Southern Medical University, Guangzhou, China; ^5^ Guangdong Provincial Key Laboratory of Proteomics, Southern Medical University, Guangzhou, China

**Keywords:** endothelin-A receptor antagonist, BQ123, therapeutic, allergic airway inflammation, group 2 innate lymphoid cell

## Abstract

Allergic airway inflammation is a universal airway disease that is driven by hyperresponsiveness to inhaled allergens. Group 2 innate lymphoid cells (ILC2s) produce copious amounts of type 2 cytokines, which lead to allergic airway inflammation. Here, we discovered that both peripheral blood of human and mouse lung ILC2s express the endothelin-A receptor (ETAR), and the expression level of ETAR was dramatically induced upon interleukin-33 (IL-33) treatment. Subsequently, both preventive and therapeutic effects of BQ123, an ETAR antagonist, on allergic airway inflammation were observed, which were associated with decreased proliferation and type 2 cytokine productions by ILC2s. Furthermore, ILC2s from BQ123 treatment were found to be functionally impaired in response to an interleukin IL-33 challenged. And BQ123 treatment also affected the phosphorylation level of the extracellular signal-regulated kinase (ERK), as well as the level of GATA binding protein 3 (GATA3) in activated ILC2s. Interestingly, after BQ123 treatment, both mouse and human ILC2s *in vitro* exhibited decreased function and downregulation of ERK signaling and GATA3 stability. These observations imply that ETAR is an important regulator of ILC2 function and may be involved in ILC2-driven pulmonary inflammation. Therefore, blocking ETAR may be a promising therapeutic strategy for allergic airway inflammation.

## Introduction

Hyperresponsive allergic inflammation is one of the most common chronic respiratory diseases worldwide and has been the most frequently diagnosed chronic disease among children in the past decade (1–5). Allergic inflammation is often defined as a reversible airway obstruction, which can also lead to immunological imbalance, inflammation of the airway mucosa, and eventually irreversible lung function impairment (6). The majority of patients exhibit an overexpression of genes involved in type 2 inflammatory pathways (7–10). Emerging evidence suggests that type 2 inflammation mainly involves the infiltration of lymphocytes, especially eosinophils, into the lung (8–11). Regrettably, patients show a poor quality of life and a high risk of mortality (1, 2). In addition, there is still no effective cure for this disease owing to its complex pathophysiology (2, 4). Therefore, understanding the underlying cellular and molecular mechanisms of pathogenesis could lay a theoretical foundation for developing novel therapeutic strategies for allergic airway inflammation.

Group 2 innate lymphoid cells (ILC2s), a subset of innate immune cells, are known to play critical roles in type 2 inflammation, protection against infection, and metabolic and tissue homeostasis (12–17). Notably, ILC2s contribute to inflammation by enhancing the activity of CD4 T helper 2 (Th2) cells and eosinophils as well as the cytokines produced by these cells (16–19). Interleukin (IL)-25, IL-33, and thymic stromal lymphopoietin are essential cytokines secreted by epithelial cells that are sensed by ILC2s (12–14). After sensitization to an allergen, this drives type 2 responses, ILC2s produce and secrete type 2 cytokines, such as IL-5 and IL-13, which in turn lead to the migration and infiltration of eosinophils and may ultimately result in the hyperresponsiveness of the airways (12–20). GATA binding protein 3 (GATA3) is considered the main transcriptional factor expressed by ILC2s (20–24). Additionally, RAR-related orphan receptor gamma t (RORγt), growth factor independent 1 transcription repressor (Gfi1), B-cell lymphoma/leukemia 11B (Bcl11b), and T cell factor-1 (TCF-1) contribute to the regulation of ILC2 function (21–24). Moreover, it has been reported that ILC2s express intercellular cell adhesion molecule-1 (ICAM-1), IL33 receptor (ST2), killer cell lectin-like receptor subfamily G member 1 (KLRG1), prostaglandin D2 receptor 2 (CRTH2), and inducible T cell costimulator (ICOS) (12, 17–19, 21–23). ILC2s also express cysteinyl leukotriene receptor 1 (CysLT1R), and leukotrienes promote ILC2 activation, which can be blocked by a CysLT1R antagonist (25–28). Besides, CD200R mediates ILC2 regulation in asthma, and can be blocked in the same manner (29). Hence, blocking receptors expressed by ILC2 might potentially inhibit ILC2 function in allergic airway inflammation.

Endothelin-1 (ET-1), expressed by pulmonary endothelial cells, is an endogenous, vasoconstrictive peptide (30, 31) that can induce the overproduction of reactive oxygen species and proinflammatory cytokines (32–34). An increased number of studies have focused on the endothelin-A receptor (ETAR), especially the ETAR antagonist. For example, an ETRQβ-002 vaccine/mAb against ETAR was used to treat pulmonary arterial hypertension and no significant immune-mediated damage was detected in vaccinated animals (35). Bosentan attenuates cigarette smoke-induced endothelin receptor (EDNR) overexpression and intrapulmonary artery tension (36). Another ETAR antagonist BQ123 is used to treat cardiovascular diseases, such as hypertension, diabetic retinopathy, and ischemia/reperfusion injury (37–40). Moreover, BQ123 relieves chronic airway inflammatory obstructive asthma (COA) (41) and acute inflammatory diseases (42). Therefore, BQ123 may be a promising therapeutic target in asthma; however, this needs to be investigated further in future studies.

Here, we show that ETAR is expressed by ILC2s from both peripheral blood human and mouse lung. BQ123, an ETAR antagonist, reduced the activation and proliferation of ILC2s and inhibited type 2 cytokine production by ILC2s. Moreover, we discovered both the preventive and therapeutic roles of BQ123 in asthma *via* ILC2s. Mechanistic studies showed that GATA3 and ERK signaling pathways are involved in the inhibition of ILC2 function driven by BQ123. Our results revealed that ETAR may be an important regulator of ILC2 function, thus providing novel insights into its role in ILC2-driven lung inflammation. In addition, BQ123 is a promising treatment option for allergic airway inflammation.

## Materials and Methods

### Mice

Female C57BL/6J mice (6 weeks old) were purchased from the Laboratory Animal Center of Southern Medical University. Rag2-deficient mice (B6.129-Rag2tm1) were purchased from the Cavens Biogel (Suzhou, China) Model Animal Research Co. Ltd., and the mice were bred in our facility at the Southern Medical University. NCG mice (NOD/ShiLtJGpt-P*rkdc* ^em26Cd52^ *Il2rg*e^m26Cd22^/Gpt) were purchased from GemPharmatech Co. Ltd. (Nanjing, China); Stock# T001475). All mice were housed in pathogen-free facilities under the following conditions: temperature, 25 ± 2°C; humidity, 50%–55%; and a 12 h light/dark cycle. All mice had free access to standard rodent pellet food and tap water according to the Southern Medical University guidelines. All experimental procedures in this study were approved by the Institutional Animal Care and Use Committee of the Southern Medical University Experimental Animal Ethics Committee (L2018166).

### Reagents and Antibodies

The reagents and antibodies used in this study are listed in **Supplementary Tables 1**, **2** respectively.

### Human Subjects

All blood samples were collected from healthy human donors at the Third Affiliated Hospital of Southern Medical University (Guangzhou, China). All subjects were screened for serum hepatitis B surface antigen, hepatitis C virus antibody, hepatitis D virus antigen, hepatitis D virus antibody, and human immunodeficiency virus antibody, and individuals positive for any of these were excluded from the study. The corresponding research plan was approved by the Biomedical Ethics Committee of the Third Affiliated Hospital of Southern Medical University. Informed consent was obtained from all donors or legal guardians at the time of admission.

### Measurement of Lung Function and Isolation of Mononuclear Cells From Lung Tissues

Lung function was assessed by lung resistance and dynamic compliance in anesthetized tracheostomized mice, *via* the Electro-Medical Measurement Systems (EMMS) and EMMS Data Acquisition software (Electromedsys supporting sciences Co Ltd, UK), and mice were synchronously challenged with aerosolized increasing doses of methacholine (0, 5, 10, 25, 50 mg/ml). Then, the experimental protocol for tissue preparation was described previously by Chen et al. (42). Briefly, the trachea was cannulated and the lungs were lavaged twice with 1 mL ice-cold PBS to collect BALF cells. Transcardial perfusion of the lungs with 20 mL cold PBS through the right ventricle of the heart was then performed to remove red blood cells, and the lungs were fixed with 4% paraformaldehyde and harvested for histological analysis. The lung lobes were cut into small pieces using scissors, digested with 0.5 mg/mL collagenase type I (Invitrogen) in Roswell Park Memorial Institute (RPMI)-1640 medium with 10% fetal bovine serum (Biological Industries) and 1% penicillin-streptomycin (Gibco) for 1 h at 37°C, and subjected to continuous agitation in a shaker. Leukocytes were obtained using a 40/80% Percoll gradient (GE Healthcare) and density gradient centrifugation at 400×g for 25 min at 25°C. Subsequently, the middle layer was gently removed using a Pasteur pipette and washed twice with PBS. The red blood cells were lysed in ammonium-chloride-potassium (ACK) buffer, and cell suspensions were filtered through 70-μm cell strainers before subsequent analyses. The reagents used in this study are listed in **Supplementary Table 1**.

### Flow Cytometric Analysis

For BALF fluid collection, leukocyte populations, such as eosinophils, were stained. Approximately 1 × 10^6^ single cell suspensions of lung cells were first incubated with lineage cocktails for 50 min at 4°C. Next, staining for surface markers and fluorochrome-conjugated streptavidin particles was performed 1 h before staining with fixable viability stain reagents to exclude dead cells. For transcription factor staining, a fixation permeabilization kit was used according to the manufacturer’s instructions; cells were stained with GATA3, Ki-67, and p-ERK1/2 (Thr204/Thr187) antibodies. To measure intracellular cytokine expression, cells were isolated *ex vivo* and stimulated in complete medium with 50 ng/mL propidium monoazide, 1 µg/mL ionomycin, and 1 µg/mL brefeldin A (BFA) for 4 h. The cells were subsequently surface-stained using IL-5 and IL-13 antibodies, fixed, and permeabilized using a CytoFix/Perm solution. All the cells were protected from light during the staining process. A LSR Fortessa flow cytometer (BD Bioscience) was used for flow cytometry data acquisition, and the data were analyzed using FlowJo v.10.0.7. Flow cytometry was conducted at the Department of Immunology at the School of Basic Medical Sciences, Southern Medical University. The mouse lung ILC2s were defined by the lack of classical lineage markers (CD3e, CD4, CD5, CD8a, CD45R/B220, CD11b, Ly-6G, CD11c, NK1.1, TER-119, TCRβ, and TCRγδ) and presence of CD45, CD127, CD90.2, CD25, and ST2. The human ILC2s were defined by the lack of lineage markers (CD2, CD3, CD8, CD14, CD16, CD19, CD34, CD56, CD235a, CD123, TCRαβ, TCRγδ, FCϵR1a, CD11b, and CD11c) and presence of CD45, CD127, and CRTH2. The gating strategy for mouse ILC2s was CD45^+^ CD4^-^ lineage^-^ CD127^+^ CD90.2^+^ CD25^+^ and that for human ILC2s was CD45^+^ lineage^-^ CD127^+^ CRTH2^+^. The antibodies used in this study are listed in **Supplementary Table 2**. Examples of ILC2 gating strategies are provided in **Supplementary Figures 1, 2**.

### ILC2s Isolation

Peripheral blood mononuclear cells (PBMCs) from healthy human or mononuclear cells from mouse lung were firstly sorted using an EasySep™ human/mouse Pan-ILC Enrichment Kit (StemCell, Canada) according to the manufacturer’s instructions. Then the total ILCs were labeled with anti-human CRTH2 or anti-mouse ST2 antibodies (eBioscience, USA), and sorted using an EasySep™ human/mouse PE Positive Selection Kit II (StemCell, Canada). Finally sorting efficiency of ILC2 was tested (**Supplementary Figure 3**).

### Lung Inflammation Models

Murine airway inflammation was induced as previously described by Monticelli et al. (24, 43). For the preventive model, such as the papain-induced pneumonia acute mouse model, the mice were anesthetized, followed by intranasal administration of papain (20 μg papain in 40 μL PBS, daily) intraperitoneally with or without BQ123 (5 mg/kg/day in 200 μL 1‰ dimethyl sulfoxide/PBS) for five consecutive days. For the IL-33 induced allergic inflammation model, six-week-old C57BL/6J or Rag2 KO mice were intranasally administered carrier-free recombinant mouse IL-33 (0.5 ug in 40 μL PBS per mouse) intraperitoneally with or without BQ123 over three consecutive days. For *A*. *alternata* experiments, mice were intranasally administered *A. alternata* (100 μg in 40 μL PBS per mouse) in the presence or absence of BQ123 on four consecutive days. For therapeutic models, six-week-old C57BL/6J or Rag2 KO mice were challenged intranasally with recombinant mouse (rm)IL-33 (0.5 μg) on days 1–3. Subsequently, the mice were treated intraperitoneally with BQ123 or PBS control for three days. Twenty-four hours after the final treatment, the mice were euthanized by cervical dislocation under isoflurane anesthesia, and the lungs and BALF were collected for analysis.

### Cytokine Detection

BALF samples of female C57BL/6J mice or Rag2 KO mice with acute lung inflammation or ILC2 co-culture supernatants were analyzed for the presence of cytokines using Thermo Scientific Multiskan FC systems. The quantity of mouse/human IL-5 and IL-13 was detected using enzyme-linked immunosorbent assay (ELISA) kits (eBioscience); the absorbance of the samples was measured using a plate reader, and the amounts were calculated from the standard curve reported as pg/mL using ELISA software.

### Histological Analysis of the Lungs

Lung tissues were fixed in 4% paraformaldehyde for 24 h. After fixation, the lungs were embedded in paraffin, cut into 4-mm-thick sections, and stained with H&E. Inflammation scores were determined as previously described by Mckay et al. (44). Briefly, the following semiquantitative scoring was used: 0–4: 0, none; 1, mild; 2, moderate; 3, marked; and 4, severe. An increment of 0.5 was used when the inflammation fell between two levels (29, 45). Besides, infiltrating cells numbers and the epithelial thickness of each individual airway were also measured as reported previously (29).

### ILC2 Function Analysis *In Vitro*

#### Mouse ILC2 Function

Purified murine ILC2s from the lungs at an initial density of approximately 5.0 x 10^3^ cells/200 µL per well (96-well round-bottom plates) in RPMI 1640 supplemented with 10% heat-inactivated fetal bovine serum were stimulated with rmIL-2 (10 ng/mL), rmIL-7 (20 ng/mL), and/or rmIL-33 (20 ng/mL) in the presence or absence of BQ123 (100 µM), ET-1 (500 ng/mL), U0126 (20 µM) or MG132 (20 µM) for 72 h. The cells were harvested to evaluate the cytokine levels by ELISA. The levels of cytokines in ILC2s (IL-5^+^ IL-13^+^) and those of transcription factor GATA3 were analyzed by flow cytometry on day 3.

#### Human ILC2 Function

PBMCs were isolated from healthy individuals as described previously by Shi et al. (46). After cell sorting, purified human ILC2s were stimulated (at an initial density of approximately 5.0 × 10^3^ cells/200 µL in 96-well round-bottom plates) with recombinant human (rh)IL-2 (20 ng/mL), rhIL-7 (20 ng/mL), and/or rhIL-33 (20 ng/ml) in the presence or absence of increasing doses of BQ123 (100 µM), ET-1 (500 ng/mL), U0126 (20 µM) or MG132 (20 µM) for 3 days. The cells were harvested to evaluate cytokine levels by ELISA. The expression level of GATA3 was analyzed by flow cytometry on day 3.

### Adoptive Transfer of ILC2s

Mouse ILC2s were directly purified from IL-33-challenged WT mice with PBS or BQ123 treatment. Human ILC2s were firstly purified from PBMCs of healthy donors, then ex-vivo treated with PBS or BQ123 in the presence of rhIL-2, rhIL-7 and rhIL-33 for 72 hours. Subsequently, approximately 4.0 × 10^4^ ILC2s in 200 µL complete medium were adoptively transferred intravenously into recipient NCG mice (12). Mice were then challenged with IL-33 intranasally for three consecutive days and were analyzed 24 h after the last challenge.

### Real-Time Quantitative Reverse Transcription-polymerase Chain Reaction (qRT-PCR)

Gene expression was quantified at the mRNA level by quantitative real-time PCR (qPCR) using the QuantStudio 6 Flex system (Thermo Fisher Scientific) and a RealStar Green Power Mixture kit (GenStar). Briefly, RNA was extracted from the lung tissue using TRIzol reagent. RT-PCR was performed using a ProFlex PCR system (ThermoFisher Scientific) with a StarScript II First-Strand cDNA Synthesis Kit (GenStar). The mRNA levels of specific genes were determined using the relative standard curve method; β-actin was used for normalization and the lowest expression level in the control group was artificially set to 1. The primer sequences are listed in **Supplementary Table 3**.

### Statistical Analysis

Experiments were repeated at least three times (n=4 each), and the data are representative of two or three independent experiments. Two-tailed Student’s t-test and one-way analysis of variance (ANOVA) were used to determine statistical significance using Prism Software 8.0 (GraphPad Software Inc.). Non-parametric Mann-Whitney tests were used to compare the differences among more than two groups when the variances were significantly different. The results are expressed as the mean ± standard error of the mean (SEM); * indicates P values, and ns denotes not significant. Differences were considered statistically significant if the P value was < 0.05. *P < 0.05; **P < 0.01; ***P < 0.001; ****P < 0.0001.

## Results

### BQ123 Exhibited Protective Effects Against Alternaria Alternata-Induced Airway Inflammation

**Figure 1:**
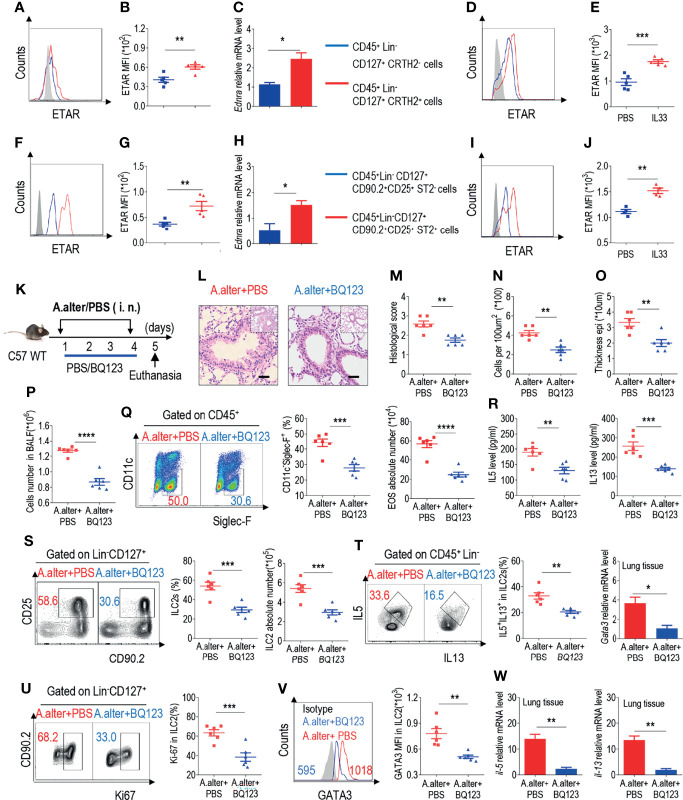
BQ13 exhibited protective effects against Alternaria alternata-induced airway inflammation. **(A)** Representative the mean fluorescence intensity (MFI) from human ILC2s of ETAR-expressing CD45^+^ Lin^-^ CD127^+^ CRTH2^-^ and CD45^+^ Lin^-^ CD127^+^ CRTH2^+^ cells (n = 5). **(B)** Statistical analysis of ETAR expression. **(C)** mRNA expression levels of human endothelin receptor A (*Ednra*) were evaluated; β-actin level was used for normalization, and the lowest expression level in *Ednra*-negative cells was artificially set to 1 (n = 3). Purified ILC2s from mouse lung and human PBMCs were cultured with rm/rh-IL-2, rm/rh-IL-7 and with or without rm/rh-IL-33 for 72 h. Then ETAR expression levels were analyzed by flow cytometry. Representative MFI **(D, I)** and statistical analysis **(E, J)** of ETAR expression were shown. **(F)** Representative results of flow cytometry MFI from mouse lung ILC2s of ETAR-expressing CD45^+^ CD4^-^ Lin^-^ CD127^+^ CD90.2^+^ CD25^+^ ST2^-^ and CD45^+^ CD4^-^ Lin^-^ CD127^+^ CD90.2^+^ CD25^+^ ST2^+^ cells (n = 5). **(G)** Representative statistical analysis of ETAR expression. **(H)** mRNA expression levels of mouse *Ednra* were evaluated; β-actin level was used for normalization, and the lowest expression level in *Ednra*-negative cells was artificially set to 1 (n = 3). **(K)** Experimental scheme. Female C57BL/6J mice were intranasally challenged with *A. alternata* on days 1–4 and were sacrificed 24 h after the last challenge on day 5. **(L–O)** Representative hematoxylin and eosin (H&E) staining of lung sections **(L)** and inflammation scores **(M)**, as well as the infiltrating cells **(N)** and airway epithelium thickness **(O)** were shown. Absolute number of BALF **(P)**, typical example of flow cytometry (left) and statistical results (right) both population and the absolute numbers of EOS in the bronchoalveolar lavage fluid (BALF) **(Q)** were indicated. **(R)** IL-5 and IL-13 levels in BALF were determined. **(S–V)** Representative results of flow cytometry, statistical analysis of the frequencies of ILC2s and absolute counts **(S)**, IL-5^+^ IL-13^+^ ILC2s **(T)**, Ki67^+^ ILC2s **(U)**, and levels of GATA3 **(V)** in the lungs were shown. **(W)** The mRNA expression levels of ILC2-related target genes in lung tissues, including *Il5, Il13*, and *Gata3*, were evaluated; *β-actin* level was used for normalization, and the lowest expression level in the *A. alternata* + BQ123 group was artificially set to 1 (n = 3). Data are representative of two or three independent experiments (n = 6 for the *A. alternata* + PBS group; n = 6 for the *A. alternata* + BQ123 group). *P < 0.05; **P < 0.01; ***P < 0.001; ****P < 0.0001. In all panels, individual results and mean ± standard error of the mean (SEM) are shown; statistical significance was determined using a two-tailed unpaired Student’s t-test **(B, C, E, G, H, J, M–U, W)** or Mann-Whitney test **(V)**.

ILC2s have been recently recognized for their central role in both initiating and perpetuating asthma-related lung inflammation (14–20). In addition, our group recently reported that the ETAR antagonist BQ123 attenuates papain-induced pneumonia (42). Therefore, in this study, we explored the relationship between ILC2s and BQ123 in pneumonia. In accordance with our published data, BQ123 attenuated the disease phenotype of papain-induced acute pneumonia, including a decrease of eosinophil infiltration and type 2 effector cytokine levels (**Supplementary Figures 4A–E**). Besides, a decrease was also observed in the population and proliferation of ILC2s, secretion of cytokines by ILC2s, and levels of transcription factor GATA3 (**Supplementary Figures 4F–I**). Similar results were observed at the mRNA level for *gata3* (**Supplementary Figure 4J**). To investigate the effect of BQ123 on ILC2s, we first examined the expression pattern of ETAR on the surface of ILC2s isolated from healthy control subjects. Results showed that ETAR expressed in ILC2s from human PBMCs at both the protein (**Figures 1A, B** and **Supplementary Figure 4K**) and mRNA levels (**Figure 1C**). The same result was observed from mouse lung ILC2s (**Figures 1F–H** and **Supplementary Figure 4L**). Moreover, ex-vivo stimulation with IL-33 of mice ILC2 and human ILC2 significantly induced the expression of ETAR in ILC2. (**Figures 1D, E, I, J**). We next evaluated the expression level of ETAR in adaptive lymphocytes and endothelial cells. Abundant expression level of ETAR in endothelial cells was observed as expected (31, 33, 35–37), and compared with other adaptive lymphocytes, higher expression level of ETAR in ILC2 was also presented (**Supplementary Figure 4M**). Collectively, these results suggest that ETAR was expressed in both human and mouse ILC2s.

Next, a clinically relevant natural allergen, *A. alternata*, which is known to trigger a type 2 innate immune response similar to that observed in clinical asthma, was used to explore the anti-inflammatory effects of BQ123 (**Figure 1K**) (47). Similar to that observed in the papain model, BQ123 attenuated the disease phenotype, resulting in a reduced histological score, decreased infiltrating cells number and airway epithelium thickness (**Figures 1L–O**) and proportion and number of eosinophils in bronchoalveolar lavage fluid (BALF) (**Figures 1P, Q** **)**. Subsequently, a decrease in IL-5 and IL-13 levels in BALF was also observed (**Figure 1R**). ILC2 (CD45^+^CD4^-^Lin^-^CD127^+^CD90.2^+^CD25^+^) and IL-5^+^ IL-13^+^ ILC2 populations were decreased in the BQ123-treated group (*A. alternata* combined with BQ123) compared with those in the phosphate-buffered saline (PBS)-treated group (*A. alternata* combined with PBS) (**Figures 1S, T**). Meanwhile, intranuclear Ki67 and GATA3 protein levels were lower in the BQ123-treated group (**Figures 1U, V**) compared with PBS-treated group. In line with the reduced protein levels, treatment with BQ123 led to decreased *Il5*, *Il13*, and *Gata3* mRNA levels (**Figure 1W**). Taken together, these results indicate that BQ123 causes remission of *A. alternata*-induced allergic lung inflammation.

### BQ123 Inhibited ILC2 Function in Response to IL-33 Challenge

**Figure 2:**
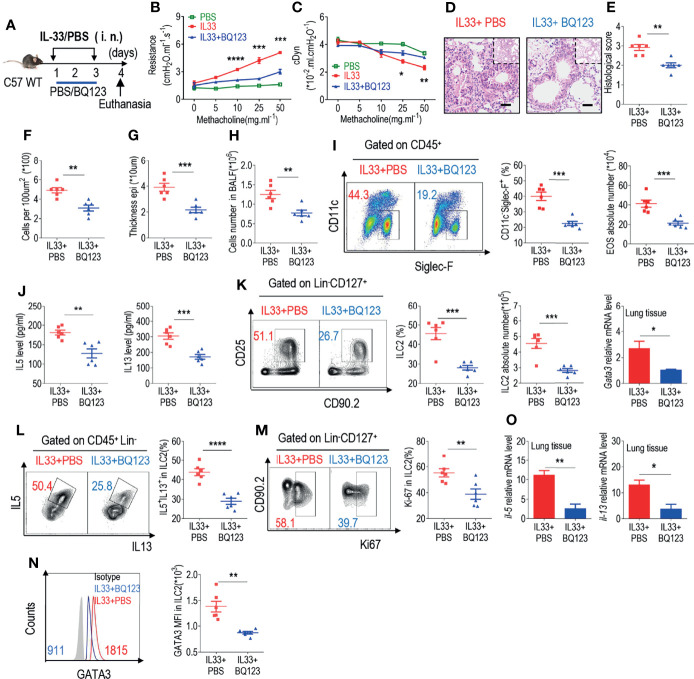
BQ123 inhibited the functional activation of ILC2s in response to IL-33 challenge. **(A)** Experimental scheme. Female C57BL/6J mice were intranasally challenged with IL-33 on days 1–3 and were sacrificed 24 h after the last challenge on day 4. **(B, C)** Line graphs show lung resistance and dynamic compliance (cDyn) in response to increasing doses of methacholine. **(D–G)** Representative hematoxylin and eosin (H&E) staining of lung sections **(D)** and inflammation scores **(E)**, as well as the infiltrating cells **(F)** and airway epithelium thickness **(G)** were presented. Absolute number of BALF **(H)**, both flow cytometry and statistical results of population, and the absolute number of EOS in the BALF **(I)** were shown. **(J)** Amounts of IL-5 and IL-13 in the BALF. **(K–N)** Representative results of both flow cytometry and statistical analysis of the frequencies of ILC2s, and absolute counts **(K)**, IL-5^+^ IL-13^+^ ILC2s **(L)**, Ki67^+^ ILC2s **(M)**, and levels of GATA3 protein **(N)** in the lungs were indicated. **(O)** The mRNA expression levels of ILC2-related target genes in lung tissues, including *Il5, Il13*, and *Gata3*, were evaluated; *β-actin* level was used for normalization, and the lowest expression level in the IL33 + BQ123 group was artificially set to 1 (n = 3). Data are representative of two or three independent experiments (n = 6 for the IL33 + phosphate-buffered saline (PBS) group; n = 6 for the IL33 + BQ123 group). *P < 0.05; **P < 0.01; ***P < 0.001; ****P < 0.0001. In all panels, individual results and mean ± standard error of the mean (SEM) are shown; statistical significance was determined using a two-tailed unpaired Student’s t-test **(B, C, E–M, O)** or Mann-Whitney test **(N)**.

The epithelial cell-derived cytokine IL-33 is known to play a crucial role in the activation of ILC2s during allergic inflammation (12, 14–17). IL-33 was intranasally administered for 3 consecutive days to induce airway inflammation (**Figure 2A**). Lung resistance and dynamic compliance was firstly assessed, as anticipated, administration with IL-33 significantly increased lung resistance (**Figure 2B**); however, lung resistance in IL33+BQ123-treated group was significantly reduced, suggesting that BQ123 engagement can prevent IL-33-induced allergic inflammation (**Figure 2B**). In agreement with these findings, dynamic compliance showed an improved response in IL33+BQ123-treated group, compared with IL33+PBS-treated group (**Figure 2C**). Subsequently, histological analysis of the lungs demonstrated that IL-33 together with BQ123, decreased inflammation index, including histological score, the epithelium thickness and inflammatory cells (**Figures 2D–G**).Similar to the results obtained in the *A. alternata* model, BQ123 inhibited eosinophil infiltration (**Figures 2H, I** **)** and decreased type 2 effector cytokine levels (**Figure 2J**), the ILC2 population (**Figure 2K**), the IL-5^+^ IL-13^+^ ILC2 population (**Figure 2L** and **Supplementary Figures 5A, B** **)**, and Ki-67 and GATA3 expression at the protein level (**Figures 2M, N** **)**. This trend was also observed at the mRNA level for *Il5*, *Il13*, and *Gata3* (**Figure 2O**). Additionally, the population of ST2^+^ ILC2s significantly decreased after treatment with BQ123 (**Supplementary Figure 5C**), whereas no difference was observed in the population of ILC2s undergoing apoptosis between BQ123-treated group and PBS-treated group (**Supplementary Figure 5D**). To investigate whether BQ123 affects other ILC subsets, including ILC1 and ILC3, we used an IL33-induced lung inflammation model and investigated them. Results showed that both ILC1 and ILC3 subsets in the intestine and lung were not affected by BQ123 (**Supplementary Figures 6A–E**). Overall, BQ123 suppresses lung ILC2 proliferation and functional activation in response to IL-33 challenge.

### BQ123 Exerted a Potential Therapeutic Effect on Allergic Inflammation

**Figure 3:**
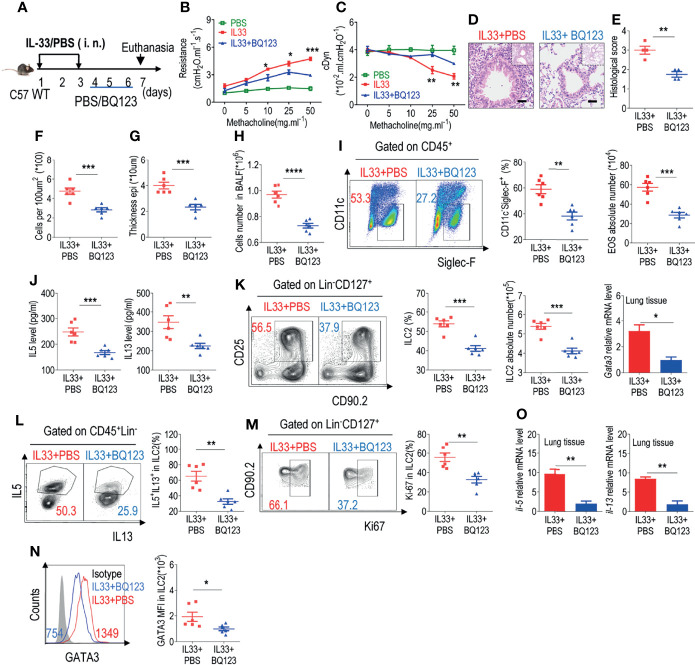
BQ123 exerted a potential therapeutic effect on allergic inflammation. **(A)** Experimental scheme. Six-week-old C57BL/6 mice were challenged intranasally with rmIL-33 (0.5 µg) on days 1–3. Subsequently, the mice were treated intranasally with BQ123 or PBS control for three days and were sacrificed 24 h after the last injection on day 7. **(B, C)** Line graphs show lung resistance and dynamic compliance (cDyn) in response to increasing doses of methacholine. **(D–G)** Representative hematoxylin and eosin (H&E) staining of lung sections **(D)** and inflammation scores **(E)**, as well as the infiltrating cells **(F)** and airway epithelium thickness **(G)** were shown. Absolute number of BALF **(H)**, typical example of flow cytometry and statistical results, the absolute number of EOS in the BALF **(I)**, and **(J)** Levels of IL-5 and IL-13 in the BALF were shown. **(K–N)** Representative results of flow cytometry and statistical analysis of the frequencies of ILC2s and absolute counts **(K)**, IL-5^+^ IL-13^+^ ILC2s **(L)**, Ki67^+^ ILC2s **(M)**, and levels of GATA3 protein **(N)** in the lungs were indicated. **(O)** The mRNA expression levels of ILC2-related target genes in lung tissues, including *Il5, Il13*, and *Gata3*, were determined; *β-actin* level was used for normalization, and the lowest expression level in the IL33 + BQ123 group was artificially set to 1 (n=3). Data are representative of two or three independent experiments (n = 6 for IL33 + PBS group; n = 6 for the IL33 + BQ123 group). *P < 0.05; **P < 0.01; ***P < 0.001; ****P < 0.0001. In all panels, individual results and mean ± standard error of the mean (SEM) are shown; statistical significance was determined using a two-tailed unpaired Student’s t-test **(B, C, E–O)**.

The administration of BQ123 during allergic inflammation exerts a significant effect on alleviation of the condition. Shafiei-Jahani et al. recently reported that CD200R engagement plays a therapeutic role in activated ILC2s (29); we further explored whether BQ123 could exert a therapeutic effect after the onset of pneumonia. The model strategy is shown in **Figure 3A**. A decrease in lung resistance and increase in dynamic compliance in IL33+BQ123-treated mice, compared with IL33+PBS treated group (**Figures 3B, C** **)** were observed, which indicate BQ123-targeted therapy ameliorates allergic inflammation, and the histological analyses further supported our conclusion (**Figures 3D–G**). Besides, BQ123 effectively reduced pulmonary inflammation through a significant decrease in eosinophils. (**Figures 3H, I** **)**, IL-5 and IL-13 levels in BALF (**Figure 3J**), ILC2 phenotypes (**Figure 3K**), and IL-5^+^ IL-13^+^ ILC2 populations (**Figure 3L**). More interestingly, Ki67 (**Figure 3M**) and GATA3 (**Figure 3N**) levels in ILC2s decreased and mRNA levels of *Il5*, *Il13*, and *Gata3* were markedly reduced in the BQ123-treated group compared with PBS-treated group (**Figure 3O**). From the above data, BQ123 plays an important role in the ILC2-driven control of allergic inflammation. To investigate whether BQ123 exerts an effect on ILC2s under physiological conditions, we next examined the inflammation index after BQ123 treatment, and same with PBS-treated group, no effect was detected under BQ123 treatment (**Supplementary Figures 7A–I**). Therefore, we concluded that BQ123 exerts a potential therapeutic effect on allergic inflammation.

### BQ123 Abrogated Allergic Lung Inflammation in a T-Cell-Independent Manner

**Figure 4:**
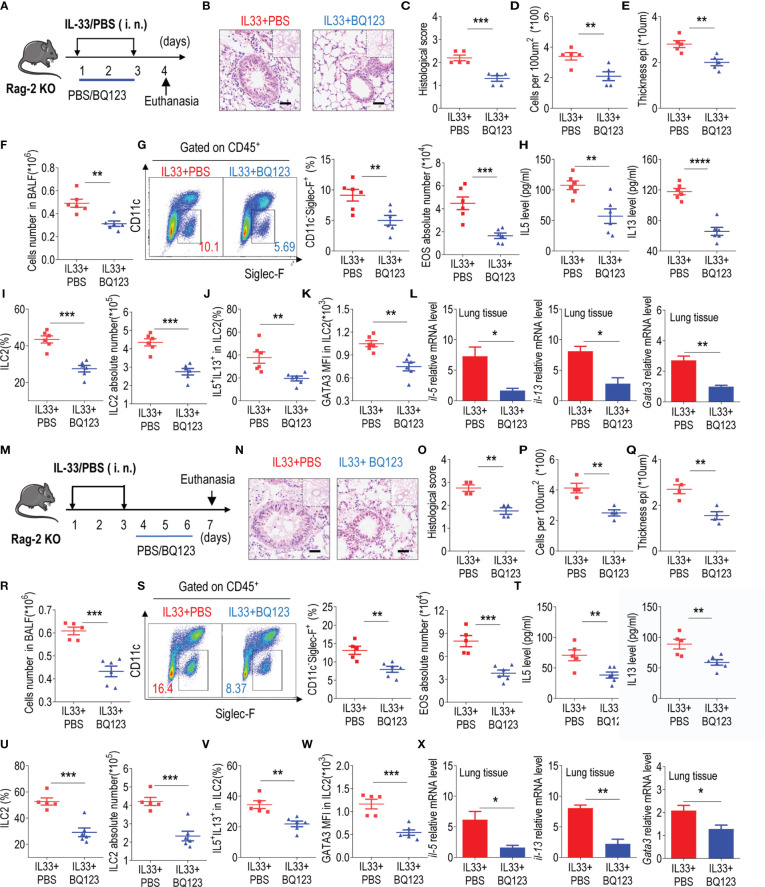
BQ123 abrogated allergic lung inflammation in a T-cell-independent manner. **(A, M)** Experimental scheme. **(B–E, N–Q)** Representative hematoxylin and eosin (H&E) staining of lung sections and inflammation scores, as well as the infiltrating cells and airway epithelium thickness were shown. **(F, R)** Absolute number of BALF. **(G, S)** Typical example of flow cytometry (left) and statistical results (right) of population and the absolute number of EOS in the BALF were shown. **(H, T)** Levels of IL-5 and IL-13 in BALF. **(I, J, U, V)** the frequencies of ILC2s and absolute counts **(I, J)**, IL-5^+^ IL-13^+^ ILC2s **(U, V)**. **(K, W)** GATA3 protein level in lung ILC2s were presented. **(L, X)** mRNA expression levels of ILC2-related target genes in lung tissues, including *Il5*, *Il13*, and *Gata3*, were determined; *β-actin* level was used for normalization, and the lowest expression level in the IL33 + BQ123 group was set to 1 (n = 3). Data are representative of two or three independent experiments (n = 6 for the preventive model; n = 5 for the therapeutic model). *P < 0.05; **P < 0.01; ***P < 0.001; ****P < 0.0001. Error bars show mean ± standard error of the mean (SEM); statistical significance was determined using a two-tailed unpaired Student’s t-test **(C–L, O–X)**.

Mounting evidence suggests that ILC2s, similar to Th2 cells, may significantly contribute to type 2 lung inflammation (48–50). To exclude the potential effect of Th2 cells on the regulation of ILC2 function by BQ123, recombination-activating gene 2-deficient mice (Rag-2 KO) was next included in our experiments (**Figures 4A, M** **)**. Both the prevention and treatment mice models are performed simultaneously. Firstly, the inflammation index data showed that BQ123 effectively alleviated lung inflammation under both models (**Figures 4B–E, N–Q**). In addition, treatment with BQ123 significantly reduced the infiltration of eosinophils (**Figures 4F, G, R, S**) and production of type 2 cytokines in BALF (**Figures 4H, T**). A significant decrease was also detected in the populations of ILC2s (**Figures 4I, U**, and **Supplementary Figures 8B, F**) and IL-5^+^ IL-13^+^ ILC2s (**Figures 4J, V**, and **Supplementary Figures 8C, G**) and protein levels of GATA3 (**Figures 4K, W**, and **Supplementary Figures 8D, H**) following BQ123 treatment. In addition, the mRNA levels of *Il5*, *Il13*, and *Gata3* decreased progressively with BQ123 treatment (**Figures 4L, X**). Collectively, these results suggest that BQ123 abrogated allergic lung inflammation in a T cell-independent manner.

### BQ123 Alleviated Airway Inflammation by Impairing ILC2 Function

**Figure 5:**
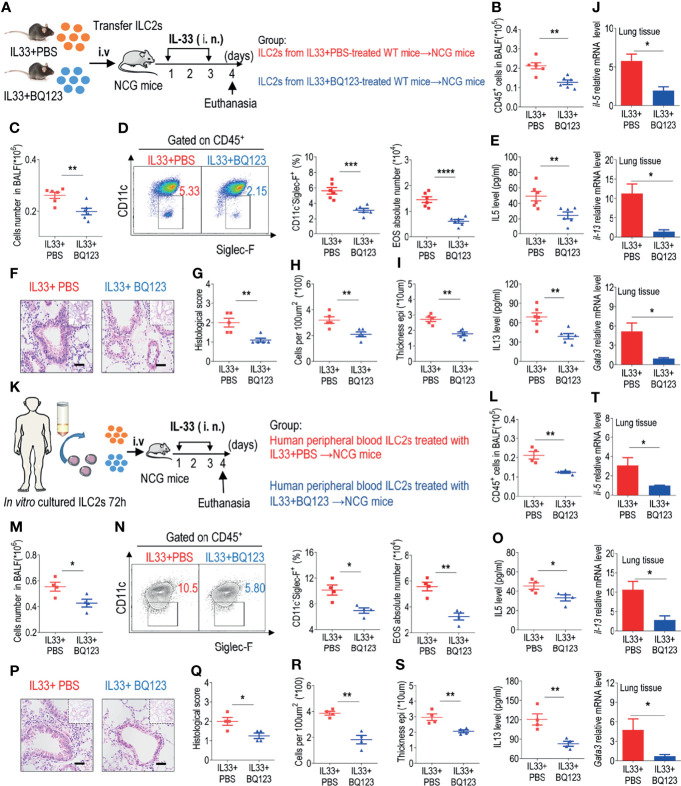
BQ123 alleviated airway inflammation by impairing ILC2 function. **(A, K)** Experimental scheme. ILC2s (approximately 5 × 10^4^ in 200 µL) were adoptively transferred intravenously into recipient NCG mice. Mice were intranasally challenged with IL-33 for three consecutive days, and the bronchoalveolar lavage fluid (BALF) and lung tissues were analyzed on day 4. **(B, L)** The number of total CD45^+^ cells in BALF. **(C, M)** Absolute number of BALF. **(D, N)** Typical example of flow cytometry (left) and statistical results (right) of population and the absolute number of EOS in the BALF. **(E, O)** IL-5 and IL-13 levels in the BALF. **(F–I, P–S)** Representative hematoxylin and eosin (H&E) staining of lung sections and inflammation scores, as well as the infiltrating cells and airway epithelium thickness were shown. **(J, T)** mRNA expression levels of *Il5*, *Il13*, and *Gata3* were evaluated; *β-actin* level was used for normalization, and the lowest expression level in the IL33 + BQ123 group was artificially set to 1. Data are representative of two independent experiments (n = 6 for the IL-33 mouse model; n = 4 for the humanized IL-33 mouse model). *P < 0.05; **P < 0.01; ***P < 0.001; ****P < 0.0001. In all panels, individual results and mean ± standard error of the mean (SEM) are shown; statistical significance was determined using a two-tailed unpaired Student’s t-test **(B–E, G–J, L–O, Q–T)**.

To further determine the specific effects of BQ123 on ILC2 function, adoptive transfer of ILC2s was performed. The same number of lung ILC2s from IL-33-challenged WT mice with or without BQ123 treatment was adoptively transferred into immunodeficient NCG mice, which lacked B cells, T cells, and ILC2s. NCG recipients were intranasally administered IL-33 (**Figure 5A**). Initially, the population of CD45^+^ immune cells in BALF (**Figure 5B**), the population of infiltrated eosinophils (**Figures 5C, D** **)**, and the levels of IL-5 and IL-13 in BALF (**Figure 5E**) were significantly lower in the BQ123-treated group than in the PBS-treated group. Then, hematoxylin and eosin (HE) staining of lung tissues revealed a distinct reduction in inflammatory infiltration in the BQ123-treated group (**Figures 5F–I**). Finally, a decrease in mRNA levels of *Il5*, *Il13*, and *Gata3* was observed (**Figure 5J**). In summary, BQ123 alleviates airway inflammation by impairing the effector function of ILC2s.

We next explored the role of BQ123 treatment in inhibiting human ILC2-mediated airway inflammation. Purified human ILC2s ex-vivo treated with PBS or BQ123 in the presence of rhIL-2, rhIL-7 and rhIL-33 for 72 hours. Then the same number of ILC2s from both group (PBS group and BQ123 group) was adoptively transferred into NCG mice, followed by intranasal administration of IL-33 for 3 consecutive days (**Figure 5K**). We firstly observed reduced the total number of CD45^+^ cells (**Figure 5L**), the number of infiltrated eosinophils in BALF (**Figures 5M, N** **)**, the amounts of IL-5 and IL-13 in BALF (**Figure 5O**). Furthermore, the attenuation of lung inflammation with decreased H&E staining inflammation scores, decreased inflammatory infiltrating cells, and weakening of the epithelium, (**Figures 5P–S**) in donors of BQ123-treated human ILC2 ex vivo was indicated, compared with PBS-treated human ILC2 ex vivo. And the mRNA level of IL-5, IL-13 and GATA3 (**Figure 5T**) were also evidenced. Collectively, these observations support that BQ123 treatment of human ILC2s can prevent airway inflammation in humanized IL-33 mouse model.

### The GATA3 Stabilization Was Impaired in BQ123-Mediated Inhibition of the ILC2s Function

**Figure 6:**
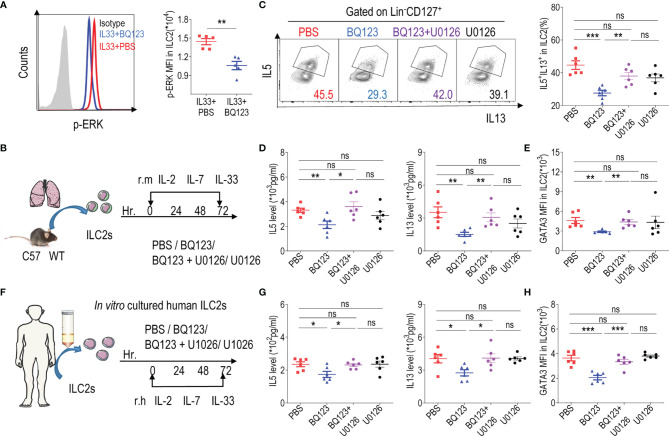
The GATA3 levels was impaired in BQ123-mediated inhibition of ILC2s function. **(A)** Representative flow cytometry results (left) and statistical analysis (right) of phosphorylated ERK levels in lung ILC2s. **(B)** Purified ILC2s from mice lung were co-cultured in the presence of rmIL-2, rmIL-7, and rmIL-33 for 72 h with BQ123 and/or the ERK inhibitor U0126. **(C)** Representative flow plots (left) and statistical results (right) of IL-5^+^ IL-13^+^ ILC2s after stimulation with PMA, ionomycin, and BFA for 4 h were shown. **(F)** Purified human peripheral blood ILC2s were co-cultured with 20 ng of rh IL-2, rhIL-7, and rhIL-33 for 72 h, with BQ123 or/and U0126. **(D, G)** Levels of IL-5 and IL-13 in co-culture supernatants were shown. **(E, H)** The intranuclear protein expression level of GATA3 was shown. Data are representative of two independent experiments (n = 5–6 for all groups). ns, not significant; *P < 0.05; **P < 0.01; ***P < 0.001. In all panels, individual results and mean ± standard error of the mean (SEM) are shown; statistical significance was determined using a two-tailed unpaired Student’s t-test **(A–E, G, H)**.

GATA3 is a transcription factor that plays crucial roles in the growth and development of ILC2s (16–18). Additionally, it has been reported that activated ERK in ILC2s affects the stability of GATA3 protein (12, 51, 52). According to our results, GATA3 protein levels were reduced with BQ123 treatment. Subsequently, the phosphorylation of ERK was examined, and results revealed that the phosphorylation of ERK (p-ERK) was significantly lower in the BQ123-treated group than in the PBS-treated group (**Figure 6A**). Therefore, we sought to determine whether ERK inhibitor U0126 alters ILC2 function *in vitro* (**Figure 6B**). In consistent with above data, the levels of IL-5 and IL-13 in cell supernatants, populations of IL-5^+^ IL-13^+^ ILC2s, and expression of GATA3 were significantly decreased in the BQ123-treated group compared with those in the PBS-treated group. As expected, U0126 strongly reversed these decreases (**Figures 6C–E**), which suggested that the ERK signaling pathway was involved in BQ123-mediated inhibition of ILC2s function. As reported by Suzuki M et al. (51), GATA3 protein is stabilized *via* the activated ERK signaling pathway. Pushing for more understand the mechanism, MG132, proteasome inhibitor was also included *in vitro* experiments. We observed that treatment with MG132 reversed the degradation of the ERK inhibitor U0126 on GATA3 protein, while this effect is weakened by BQ123 participation (**Supplementary Figure 9A**). This supported that the GATA3 stabilization was impaired in BQ123-mediated inhibition of the effector function of ILC2s. To better establish the underlying mechanisms, we next explored the effect of BQ123 on human ILC2s (**Figure 6F**). Consistent with our murine ILC2 observations, BQ123 significantly suppressed the effector function of human ILC2s by decreasing the IL-5 and IL-13 production, GATA3 expression and ILC2 phenotype, these effects were significantly reversed by U0126 (**Figures 6G, H** and **Supplementary Figure 9B**). Same to mouse data, treatment of ILC2s with U0126 and MG132 resulted in the increase of GATA3 content, whereas BQ123 participation attenuated the effect (**Supplementary Figure 9C**). Given that the effect of BQ123 on ILC2 cells may be require the presence of endothelin, the content of endothelin in culture supernatant was further investigated. Results showed that the content of endothelin was significantly increased after 3 days ex-vivo stimulation of mice ILC2 and human ILC2 (**Supplementary Figures 9D, E**), which implies another underlying mechanism of BQ123 affecting on ILC2. Taken together, these data suggest that GATA3 is a target of the ERK signaling pathway in the BQ123 inhibition of the effector function of ILC2s.

## Discussion

In the present study, we ascertained that ETAR is expressed by both human and mouse ILC2s. We also elucidated the mechanism of action, signaling pathways, and therapeutic potential of BQ123, an ETAR antagonist, using pulmonary ILC2s in the context of lung inflammation. The expression of ETAR by ILC2s during pulmonary inflammation implies the therapeutic viability of targeting ETAR.

Initially, we utilized the *A. alternata*-based model of allergic airway inflammation because *A. alternata* is commonly found in the environment and is a well-known allergen in humans (45, 53). *A. alternata* has been reported to cause allergic inflammation in mice independent of adaptive immunity, making it an ideal model for studying ILC2-dependent asthma (12, 29). We subsequently utilized an IL-33-based model of allergic airway inflammation because IL-33 and IL-25 have been previously shown to induce ILC2-mediated lung inflammation and IL-33 is more potent than IL-25 (54–56). Considering the crosstalk network between Th2 cells and ILC2s during allergic inflammation, Rag2 KO mice, which lack T and B cells, were used to induce allergic inflammation to exclude the effects of Th2 cells on amelioration of the condition (48–50, 57–60). Based on our results, we concluded that the inhibitory effect of BQ123 on ILC2s was independent of T cells (**Figures 4A–X**). In addition, adoptive transfer of ILC2s from BQ123- or PBS-treated mice into immunodeficient NCG mice further demonstrated that BQ123 is essential for the inhibition of the functions of lung ILC2s upon IL-33 administration. Overall, these observations support the hypothesis that BQ123 specifically impairs the function of ILC2s.

GATA3 is a key regulator of ILC2 development, maintenance, and function (16–18). Remarkably reduced GATA3 expression levels (at both the protein and mRNA levels) were observed in the BQ123-treated group compared with those in the PBS-treated group, suggesting that GATA3 mediates the regulatory effects of BQ123 on ILC2s. The ERK signaling pathway regulates the protein level of GATA3. We further demonstrated that BQ123 inhibited the ERK pathway in activated ILC2s, and blockage of the ERK signaling pathway abrogated the effect of BQ123 on both mouse and human ILC2s *in vitro*, suggesting that the ERK/GATA3 pathway is involved in the BQ123-mediated inhibition of ILC2 effector function.

Understanding the critical interplay between ILC2s and their respective physical tissue niches during homeostasis and/or inflammation remains an area of immense interest. Previous studies have highlighted that the ET1-ETAR axis participates in the progression of cardiovascular-related diseases in humans and mice (37–40). Here, we report that ETAR is expressed by ILC2s. The ETAR antagonist BQ123 plays a vital role in type 2 airway inflammation by negatively regulating the effector function of ILC2s. ET-1, an agonist of ETAR, is an endogenous, vasoconstrictive peptide and is reportedly expressed by pulmonary endothelial cells (61–63). We also demonstrated that ET-1 is highly expressed in both non-immune and non-ILC2s (**Supplementary Figures 10A, B**). Subsequently, we tested the effect of ET1 on ILC2s. We found that the population of ILC2 and levels of cytokines secreted by ILC2 increased significantly after ET1 protein treatment *in vitro* in both mouse and human ILC2s (**Supplementary Figures 11A–H**), which is in contrast to the results obtained following BQ123 treatment. These observations collectively reveal the importance of ETAR in ILC2-driven innate immunity during airway inflammation. The role of the ET1-ETAR axis in the function of ILC2s requires further investigation.

In general, previous studies focused on the role of BQ123 in alleviating chronic cardiovascular diseases by regulating the ET1-ETAR system, but not on its anti-inflammatory effect (30, 31, 64). It was later reported that BQ123 inhibits uric acid-induced epithelial–mesenchymal transition and reduces the levels of ET-1 and nicotinamide adenine dinucleotide phosphate oxidase 4 (NOX4) in NRK-52E cells (65). However, to the best of our knowledge, it is not yet clear whether BQ123 directly regulates immune cell functions and alleviates allergic lung inflammation. In terms of acute inflammatory disease, it is important to reverse the established inflammation, in addition to exerting preventive effects (66). Therefore, the effect of BQ123 on the immune mechanism in the treatment of inflammatory diseases needs further research. Interestingly, our study revealed that BQ123 not only plays a preventive role in the onset of ILC2-mediated lung inflammatory diseases but also exerts a therapeutic effect in models that were previously established. Collectively, these results suggest that BQ123 exerts a therapeutic effect.

Currently, there is no established ILC2-targeting therapy; the mitigation effect of ILC2 on pneumonia has mainly focused on inhibitory pathways for ILC2s in previous studies. Several groups have found that type I and II interferons (IFN-α, IFN-β, and IFN-γ) and IL-27 inhibit ILC2 functions *via* the activation of signal transducer and activator of transcription 1 (STAT1), indicating that the STAT1 pathway is a negative regulator of ILC2s (67–69). Lipid mediators (prostaglandin (PG)-E2 and PGI2) are known to activate ILC2 function by activating adenylate cyclase and cyclic AMP (cAMP)/protein kinase A (PKA) pathways (25–28). Recent studies by Chu et al. indicated that the autonomic airway fibers produce acetylcholine (Ach), which plays an important role in regulating ILC2 responses (70). Collectively, our results suggest that BQ123 negatively regulates ILC2 effector function directly, revealing a previously unrecognized therapeutic role of BQ123. In the future, this area of research may lead to the development of novel therapies for airway type 2 inflammatory diseases aimed at blocking ILC2 activators and activating inhibitory pathways.

In conclusion, our study demonstrates that ETAR is a vital regulator of ILC2 function. Moreover, the ETAR antagonist BQ123 impairs the function of ILC2s, whereas its agonist ET1 promotes the activation of ILC2s. Furthermore, we discussed the preventive and therapeutic effects of BQ123 on allergic asthma. Our findings provide avenues for designing novel therapeutics that target ILC2s to treat asthma and allergic diseases.

## Data Availability Statement

The original contributions presented in the study are included in the article/**Supplementary Material**. Further inquiries can be directed to the corresponding authors.

## Ethics Statement

The animal study was reviewed and approved by the Institutional Animal Care and Use Committee of the Southern Medical University Experimental Animal Ethics Committee.

## Author Contributions

YH and GX conceived and supervised the study and wrote the manuscript. XZ, ZC, SZ, HS, XL, XYL, ZX, MC, and JL performed the experiments. Authors with equal contributions are listed in alphabetical order. All authors contributed to the article and approved the submitted version.

## Funding

This work was supported by grants from the following institutions: the High-level Talent Start-up Funding of the Southern Medical University, the National Natural Science Foundation of China (81971420, 81991511, and 82171706), the Guangdong Special Support Program for Youth Science and Technology Innovation Talents (2019TQ05Y585), the National Natural Science Foundation of Guangdong (2019A1515011435), and the Science and Technology Program of Guangzhou (201904010073), The postdoctoral research project start-up funding of Affiliated Dongguan Hospital, Southern Medical University (294526).

## Conflict of Interest

The authors declare that the research was conducted in the absence of any commercial or financial relationships that could be construed as a potential conflict of interest.

## Publisher’s Note

All claims expressed in this article are solely those of the authors and do not necessarily represent those of their affiliated organizations, or those of the publisher, the editors and the reviewers. Any product that may be evaluated in this article, or claim that may be made by its manufacturer, is not guaranteed or endorsed by the publisher.
