# Corrigendum: Endothelin-A Receptor Antagonist Alleviates Allergic Airway Inflammation *via* the Inhibition of ILC2 Function

**DOI:** 10.3389/fimmu.2022.877694

**Published:** 2022-03-28

**Authors:** Xiaogang Zhang, Ziyang Chen, Shaowen Zuo, Hengbiao Sun, Xinyao Li, Xiao Lu, Zhe Xing, Meiqi Chen, Jingping Liu, Gang Xiao, Yumei He

**Affiliations:** ^1^ Department of Immunology, School of Basic Medical Sciences, Southern Medical University, Guangzhou, China; ^2^ Department of Neurosurgery Affiliated Dongguan Hospital, Southern Medical University, Dongguan, China; ^3^ Department of Clinical Laboratory, The Third Affiliated Hospital of Southern Medical University, Southern Medical University, Guangzhou, China; ^4^ Guangdong Provincial Key Laboratory of Single Cell Technology and Application, Southern Medical University, Guangzhou, China; ^5^ Guangdong Provincial Key Laboratory of Proteomics, Southern Medical University, Guangzhou, China

**Keywords:** endothelin-A receptor antagonist, BQ123, therapeutic, allergic airway inflammation, group 2 innate lymphoid cell

In the original article, there was a mistake in **Figure 1** as published. In **Figure 1C**, the blue line “CD45^+^ Lin^-^ CD127^+^ CRTH2^+^” should be labelled “CD45^+^ Lin^-^ CD127^+^ CRTH2^-^ cells”; in **Figure 1H**, the blue line “CD45^+^ Lin CD127^+^ CD90.2^+^ CD25^+^ ST2^-^ cells” should be labelled “CD45^+^ Lin^-^ CD127^+^ CD90.2^+^ CD25^+^ ST2^-^ cells”. The corrected **Figure 1** appears below.

**Figure 1:**
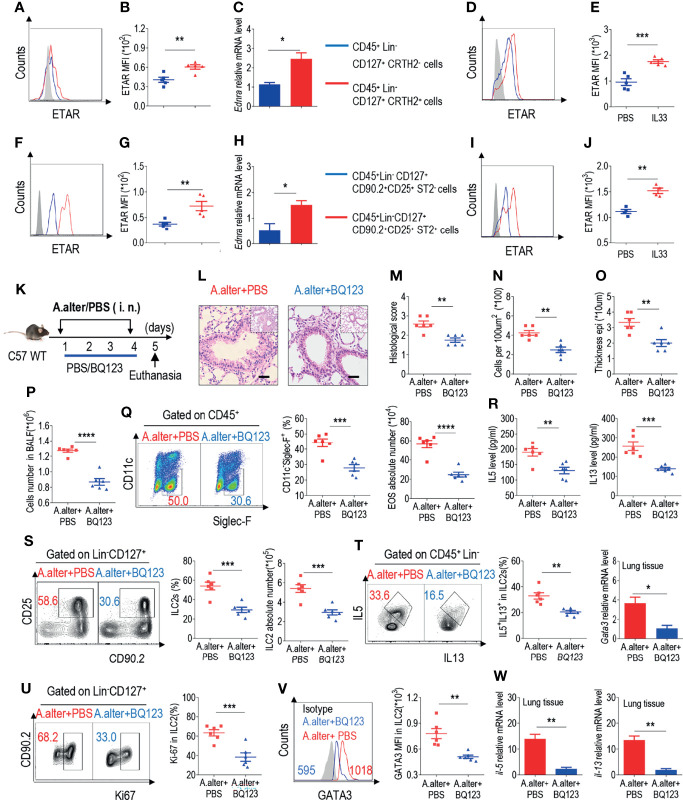
BQ123 exhibited protective effects against Alternaria alternata-induced airway inflammation **(A)** Representative the mean fluorescence intensity (MFI) from human ILC2s of ETAR-expressing CD45^+^ Lin^-^ CD127^+^ CRTH2^-^ cells and CD45^+^ Lin^-^ CD127^+^ CRTH2^+^ cells (n = 5). **(B)** Statistical analysis of ETAR expression. **(C)** mRNA expression levels of human endothelin receptor A (*Ednra*) were evaluated; *β-actin* level was used for normalization, and the lowest expression level in *Ednra*-negative cells was artificially set to 1 (n = 3). Purified ILC2s from mouse lung and human PBMCs were cultured with rm/rh-IL-2, rm/rh-IL-7 and with or without rm/rh-IL-33 for 72 h. Then ETAR expression levels were analyzed by flow cytometry. Representative MFI **(D, I)** and statistical analysis **(E, J)** of ETAR expression were shown. **(F)** Representative results of flow cytometry MFI from mouse lung ILC2s of ETAR -expressing CD45^+^ Lin^-^ CD127^+^ CD90.2^+^ CD25^+^ ST2^-^ cells and CD45^+^ Lin^-^ CD127^+^ CD90.2^+^ CD25^+^ ST2^+^ cells (n=5). **(G)** Representative statistical analysis of ETAR expression. **(H)** mRNA expression levels of mouse *Ednra* were evaluated; *β-actin* level was used for normalization, and the lowest expression level in *Ednra* -negative cells was artificially set to 1 (n = 3). **(K)** Experimental scheme. Female C57BL/6J mice were intranasally challenged with *A. alternata* on days 1–4 and were sacrificed 24 h after the last challenge on day 5. **(L–O)** Representative hematoxylin and eosin (H&E) staining of lung sections **(L)** and inflammation scores **(M)**, as well as the infiltrating cells **(N)** and airway epithelium thickness **(O)** were shown. Bars, 100μm. Absolute number of BALF **(P)**, typical example of flow cytometry (left) and statistical results (right) both population and the absolute numbers of EOS in the bronchoalveolar lavage fluid (BALF) **(Q)** were indicated. **(R)** IL-5 and IL-13 levels in BALF were determined. **(S–V)** Representative results of flow cytometry, statistical analysis of the frequencies of ILC2s and absolute counts **(S)**, IL-5^+^ IL-13^+^ ILC2s **(T)**, Ki67^+^ ILC2s **(U)**, and levels of GATA3 **(V)** in the lungs were shown. **(W)** The mRNA expression levels of ILC2-related target genes in lung tissues, including *Il5*, *Il13*, and *Gata3*, were evaluated; *β-actin* level was used for normalization, and the lowest expression level in the *A. alternata* + BQ123 group was artificially set to 1 (n = 3). Data are representative of two or three independent experiments (n = 6 for the *A. alternata* + PBS group; n = 6 for the *A. alternata* + BQ123 group). *P < 0.05; **P < 0.01; ***P < 0.001; ****P < 0.0001. In all panels, individual results and mean ± standard error of the mean (SEM) are shown; statistical significance was determined using a two-tailed unpaired Student’s t-test **(B, C, E, G, H, J, M–U, W)** or Mann-Whitney test **(V)**.

In the original article, there was a mistake in **Figure 2** as published. In **Figures 2F–G**, the x-axis labels are incorrect. The corrected **Figure 2** appears below.

**Figure 2:**
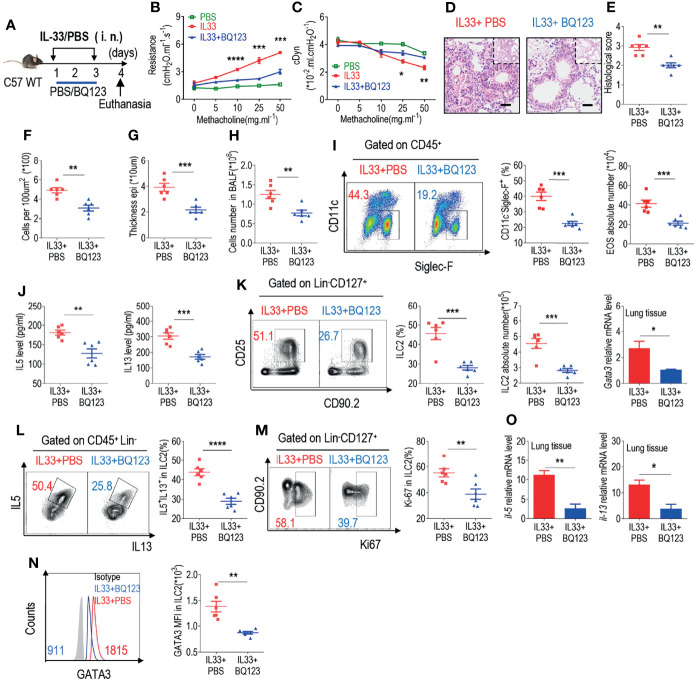
BQ123 inhibited the functional activation of ILC2s in response to IL-33 challenge. **(A)** Experimental scheme. Female C57BL/6J mice were intranasally challenged with IL-33 on days 1–3 and were sacrificed 24 h after the last challenge on day 4. **(B, C)** Line graphs show lung resistance and dynamic compliance (cDyn) in response to increasing doses of methacholine. **(D–G)** Representative hematoxylin and eosin (H&E) staining of lung sections **(D)** and inflammation scores (**E**), as well as the infiltrating cells **(F)** and airway epithelium thickness **(G)** were presented. Bars, 100μm. Absolute number of BALF **(H)**, both flow cytometry and statistical results of population, and the absolute number of EOS in the BALF **(I)** were shown. **(J)** Amounts of IL-5 and IL-13 in the BALF. **(K–N)** Representative results of both flow cytometry and statistical analysis of the frequencies of ILC2s, and absolute counts **(K)**, IL-5^+^ IL-13^+^ ILC2s **(L)**, Ki67^+^ ILC2s **(M)**, and levels of GATA3 protein **(N)** in the lungs were indicated. **(O)**The mRNA expression levels of ILC2-related target genes in lung tissues, including *Il5, Il13*, and *Gata3*, were evaluated; *β-actin* level was used for normalization, and the lowest expression level in the IL33 + BQ123 group was artificially set to 1 (n = 3). Data are representative of two or three independent experiments (n = 6 for the IL33 + phosphate-buffered saline (PBS) group; n = 6 for the IL33 + BQ123 group). Note: *P < 0.05; **P < 0.01; ***P < 0.001; ****P < 0.0001. In all panels, individual results and mean ± standard error of the mean (SEM) are shown; statistical significance was determined using a two-tailed unpaired Student’s t-test **(B, C, E–M, O)** or Mann-Whitney test **(N)**.

In the original article, there was a mistake in **Figure 3** as published. In **Figures 3F–G**, the x-axis labels are incorrect. The corrected **Figure 3** appears below.

**Figure 3:**
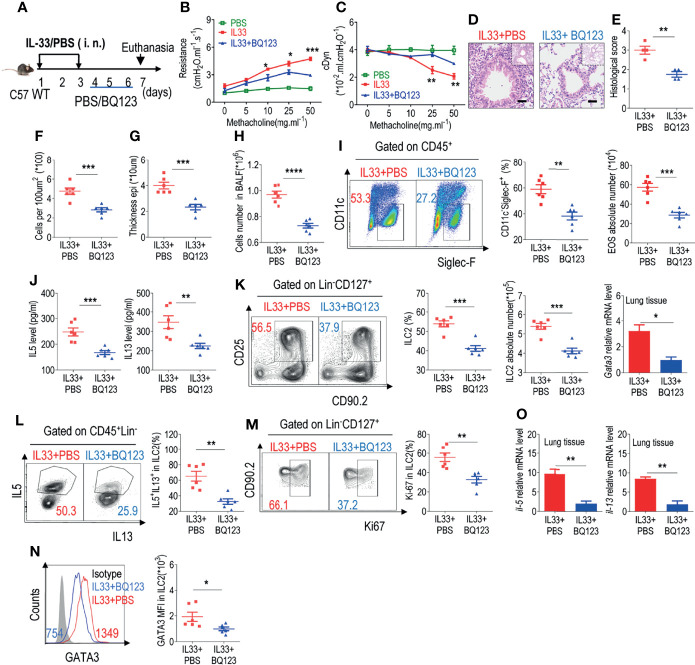
BQ123 exerted a potential therapeutic effect on allergic inflammation. **(A)** Experimental scheme. Six-week-old C57BL/6J mice were challenged intraperitoneally with rmIL-33 (0.5 µg) on days 1–3. Subsequently, the mice were treated intraperitoneally with BQ123 or PBS control for three days and were sacrificed 24 h after the last injection on day 7. **(B, C)** Line graphs show lung resistance and dynamic compliance (cDyn) in response to increasing doses of methacholine. **(D–G)** Representative hematoxylin and eosin (H&E) staining of lung sections **(D)** and inflammation scores **(E)**, as well as the infiltrating cells **(F)** and airway epithelium thickness **(G)** were shown. Bars, 100μm. Absolute number of BALF **(H)**, typical example of flow cytometry and statistical results, the absolute number of EOS in the BALF **(I, J)** Levels of IL-5 and IL-13 in the BALF were shown. **(K–N)** Representative results of flow cytometry and statistical analysis of the frequencies of ILC2s and absolute counts **(K)**, IL-5^+^ IL-13^+^ ILC2s **(L)**, Ki67^+^ ILC2s **(M)**, and levels of GATA3 protein **(N)** in the lungs were indicated. **(O)** The mRNA expression levels of ILC2-related target genes in lung tissues, including *Il5, Il13*, and *Gata3*, were determined; *β-actin* level was used for normalization, and the lowest expression level in the IL33 + BQ123 group was artificially set to 1 (n=3). Data are representative of two or three independent experiments (n = 6 for IL33 + PBS group; n = 6 for the IL33 + BQ123 group). Note: *P < 0.05; **P < 0.01; ***P < 0.001; ****P < 0.0001. In all panels, individual results and mean ± standard error of the mean (SEM) are shown; statistical significance was determined using a two-tailed unpaired Student’s t-test **(B, C, E–O)**.

In the original article, there was a mistake in **Figure 5** as published. In **Figures 5R–S**, the x-axis corner labels are incorrect. The corrected **Figure 5** appears below.

**Figure 5:**
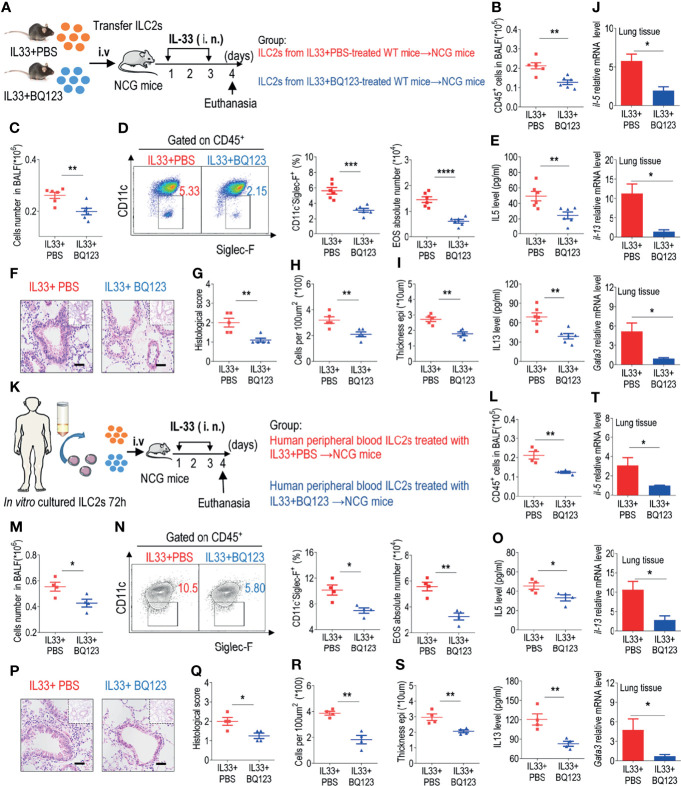
BQ123 alleviated airway inflammation by impairing ILC2 function. **(A, K)** Experimental scheme. ILC2s (approximately 5 × 10^4^ in 200 µL) were adoptively transferred intravenously into recipient NCG mice. Mice were intranasally challenged with IL-33 for three consecutive days, and the bronchoalveolar lavage fluid (BALF) and lung tissues were analyzed on day 4. **(B, L)** The number of total CD45^+^ cells in BALF. **(C, M)** Absolute number of BALF. **(D, N)** Typical example of flow cytometry (left) and statistical results (right) of population and the absolute number of EOS in the BALF. **(E, O)** IL-5 and IL-13 levels in the BALF. **(F–I, P–S)** Representative hematoxylin and eosin (H&E) staining of lung sections and inflammation scores, as well as the infiltrating cells and airway epithelium thickness were shown. Bars, 100μm. **(J, T)** mRNA expression levels of *Il5*, *Il13*, and *Gata3* were evaluated; *β-actin* level was used for normalization, and the lowest expression level in the IL33 + BQ123 group was artificially set to 1. Data are representative of two independent experiments (n = 6 for the IL-33 mouse model; n = 4 for the humanized IL-33 mouse model). Note: *P < 0.05; **P < 0.01; ***P < 0.001; ****P < 0.0001. In all panels, individual results and mean ± standard error of the mean (SEM) are shown; statistical significance was determined using a two-tailed unpaired Student’s t-test **(B–E, G–J, L–O, Q–T)**.

In the original article, there was a mistake in **Supplementary Figure 4** as published. In **Supplementary Figures 4K–L**, the line labels are incorrect. The corrected **Supplementary Figure 4** appears below.

In the original article, there was an error. The IL33 dose unit was written incorrectly.

A correction has been made to **Materials and Methods**, “*Lung Inflammation Models*”, paragraph 1:

“Murine airway inflammation was induced as previously described by Monticelli et al. (24, 43). For the preventive model, such as the papain-induced pneumonia acute mouse model, the mice were anesthetized, followed by intranasal administration of papain (20 µg papain in 40 µL PBS, daily) intraperitoneally with or without BQ123 (5 mg/kg/day in 200 µL 1‰ dimethyl sulfoxide/PBS) for five consecutive days. For the IL-33-induced allergic inflammation model, six-week-old C57BL/6J or Rag2 KO mice were intranasally administered carrier-free recombinant mouse IL-33 (0.5 ug in 40 µL PBS per mouse) intraperitoneally with or without BQ123 over three consecutive days. For *A. alternata* experiments, mice were intranasally administered *A. alternata* (100 µg in 40 µL PBS per mouse) in the presence or absence of BQ123 on four consecutive days. For therapeutic models, six-week-old C57BL/6J or Rag2 KO mice were challenged intranasally with recombinant mouse (rm)IL-33 (0.5 µg) on days 1–3. Subsequently, the mice were treated intraperitoneally with BQ123 or PBS control for three days. Twenty-four hours after the final treatment, the mice were euthanized by cervical dislocation under isoflurane anesthesia, and the lungs and BALF were collected for analysis.”

In the original article, there was an error. A letter is missing in the first sub-section header of **Results**. The corrected sub-section header is “*BQ123 Exhibited Protective Effects Against Alternaria Alternata-Induced Airway Inflammation*”.

The authors apologize for these errors and state that they do not change the scientific conclusions of the article in any way. The original article has been updated.

## Publisher’s Note

All claims expressed in this article are solely those of the authors and do not necessarily represent those of their affiliated organizations, or those of the publisher, the editors and the reviewers. Any product that may be evaluated in this article, or claim that may be made by its manufacturer, is not guaranteed or endorsed by the publisher.
