# Supplementary material for: Endothelin-A Receptor Antagonist Alleviates Allergic Airway Inflammation via the Inhibition of ILC2 Function
Source: Front Immunol. 2022 Feb 11;13:835953. doi: 10.3389/fimmu.2022.835953 (PMC8873101; doi:10.3389/fimmu.2022.835953)
Supplement: Supplementary file 1 [file DataSheet_1.pdf]

Supplementary Materials for

Endothelin-A receptor Antagonist Alleviates Allergic Airway Inflammation Via the Inhibition of ILC2 Function

The PDF file includes:

Supplementary Figure 1 to Supplementary Figure 11

Supplementary Figure 1:

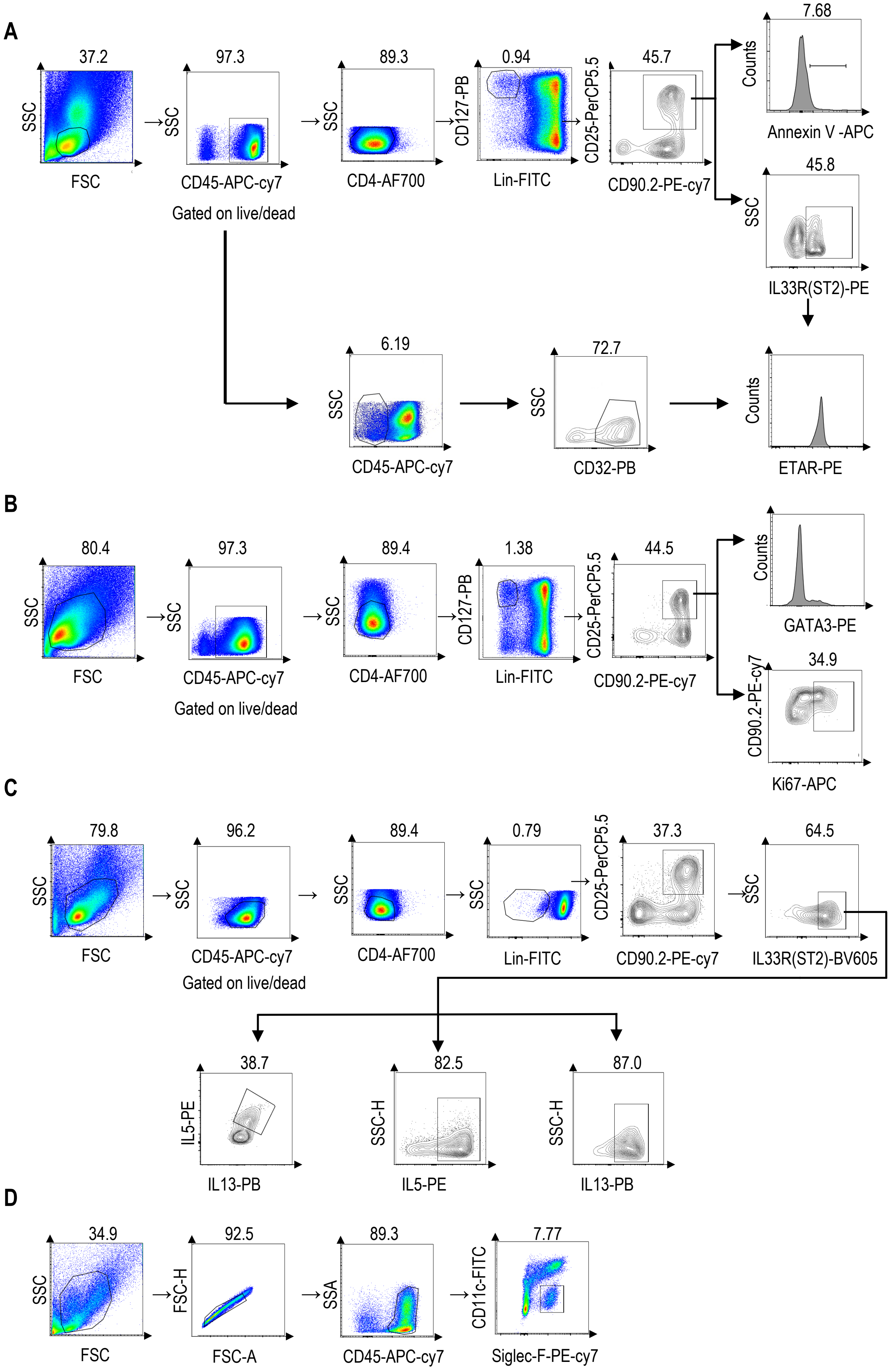

Supplementary Figure 1. Flow cytometry gating strategy in mouse experiments.

(A) Gating strategy for mouse cells expressing the Endothelin-A Receptor (ETAR) and apoptosis both ILC2s and $CD32^+$ endothelial cells. **(B)** Gating strategy for transcription factor GATA3, cell proliferation Ki67, (C) $IL\text{-}5^+$ $IL\text{-}13^+$ ILC2s ($CD45^+$ $CD4^-$ $Lin^-$$CD90.2^+$ $CD25^+$ $ST2^+$ $IL5^+$ $IL13^+$), and (D) eosinophils ($CD45^+$ $CD11c^-$ Siglec-F^+).

Supplementary Figure 2:

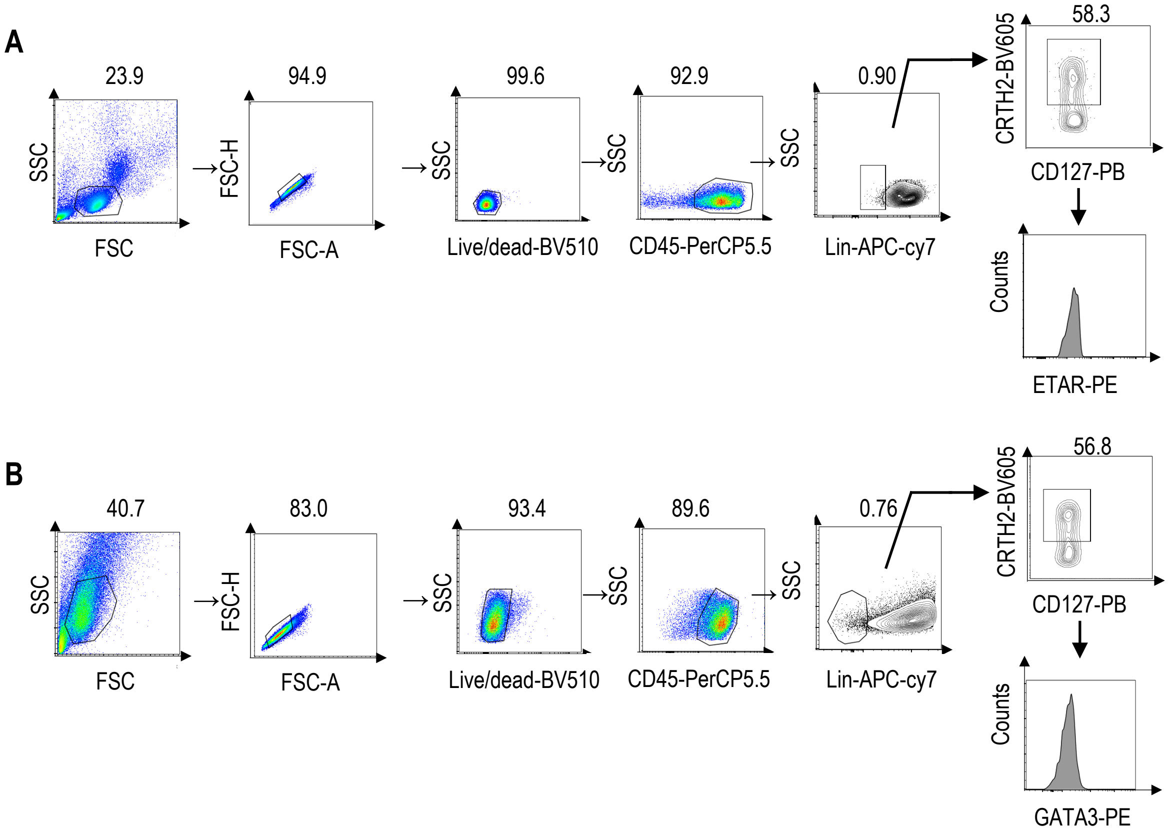

Supplementary Figure 2. Flow cytometry gating strategy in human experiments.

(A and B) Gating strategy for human group 2 innate lymphoid cells (ILC2s) expressing the Endothelin-A Receptor (ETAR) and transcription factor GATA3.

Supplementary Figure 3:

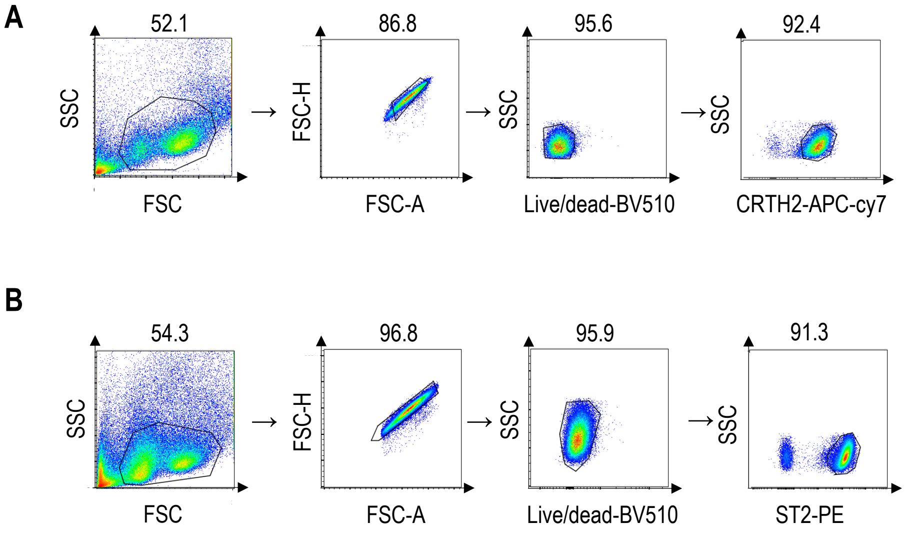

Supplementary Figure 3. Sorting efficiency of human and mouse group 2 innate lymphoid cells (ILC2s).

(A and B) Representative flow cytometry results of the sorting efficiency of ILC2s.

Supplementary Figure 4:

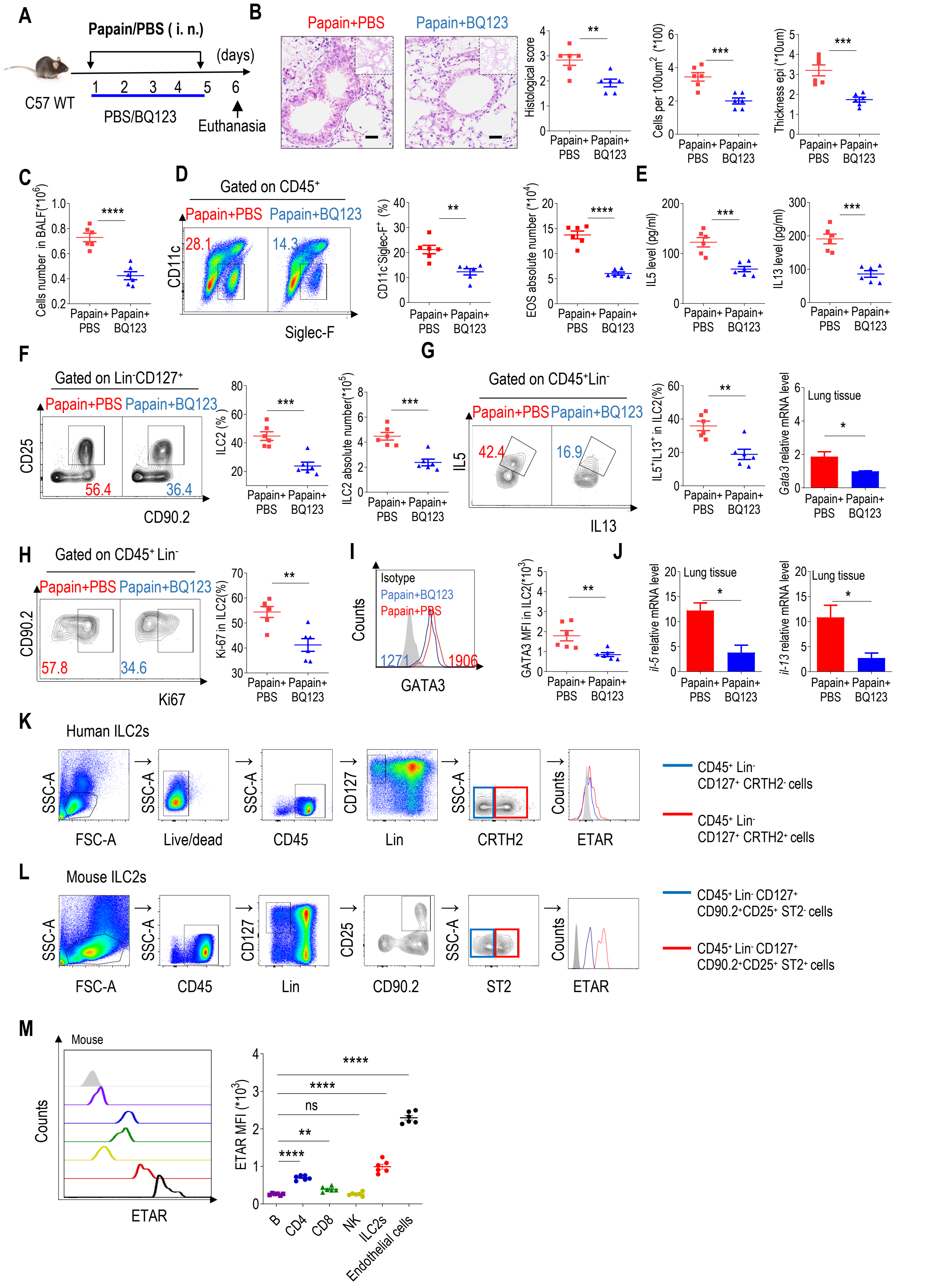

Supplementary Figure 4. Papain-induced allergic lung inflammation is relieved by BQ123 during the responses of pulmonary group 2 innate lymphoid cells (ILC2s).

(A) Female C57BL/6J mice were intranasally challenged with papain on days 1–5 and were sacrificed 24 h after the last challenge on day 6. (B) Representative hematoxylin and eosin (H&E) staining of lung sections and inflammation scores, including the infiltrating cells and airway epithelium thickness. Bars, 100μm. (C) Absolute number of BALF. (D) Typical example of flow cytometry and statistical results both population and the absolute number of EOS in the BALF. (E) Interleukin (IL)-5 and IL-13 levels in BALF. (F-I) Representative results of flow cytometry (left) and statistical analysis (right) of the frequencies of ILC2s (F)， including absolute counts (F), IL-5^{+} IL-13^{+} ILC2s (G), and Ki67^{+} ILC2s (H) and levels of GATA binding protein 3 (GATA3) (I) in the lungs. (J) mRNA expression levels of ILC2-related target genes in lung tissues, including *Il5, Il13,* and *Gata3,* were determined; *β-actin* level was used for normalization, and the lowest expression level in the papain + BQ123 group was set to 1 (n = 3). Data are representative of two independent experiments (n = 6 for papain + phosphate-buffered saline (PBS) group; n = 6 for papain + BQ123 group). (K) Representative gating strategy of human ILC2s and the mean fluorescence intensity (MFI) of ETAR-expressing CD45^{+} Lin^{-} CD127^{+} CRTH2^{-} and CD45^{+} Lin^{-} CD127^{+} CRTH2^{+} cells (n = 5). (**L**) Representative gating strategy of mouse lung ILC2s and the MFI of ETAR-expressing CD45^{+} Lin^{-} CD127^{+} CD90.2^{+} CD25^{+} ST2^{-} and CD45^{+} Lin^{-} CD127^{+} CD90.2^{+} CD25^{+} ST2^{+} cells. (M) Representative flow cytometry results of the mean fluorescence intensity (MFI) and statistical analysis of ETAR-expressing B cells, CD4 cells，CD8 cells, NK cells， ILC2 (CD45^{+} CD4^{-} Lin^{-} CD127^{+} CD90.2^{+} CD25^{+} ST2^{+}) cells, and Endothelial (CD45^{-} CD31^{+}) cells (n = 5). Note: ns, not significant; *P < 0.05; **P < 0.01; ***P < 0.001; ****P < 0.0001. In all panels, individual results and mean ± standard error of the mean (SEM) are shown; statistical significance was determined using a two-tailed unpaired Student's t test (B, C, D, E, F, G, H, I, J, and M).

Supplementary Figure 5:

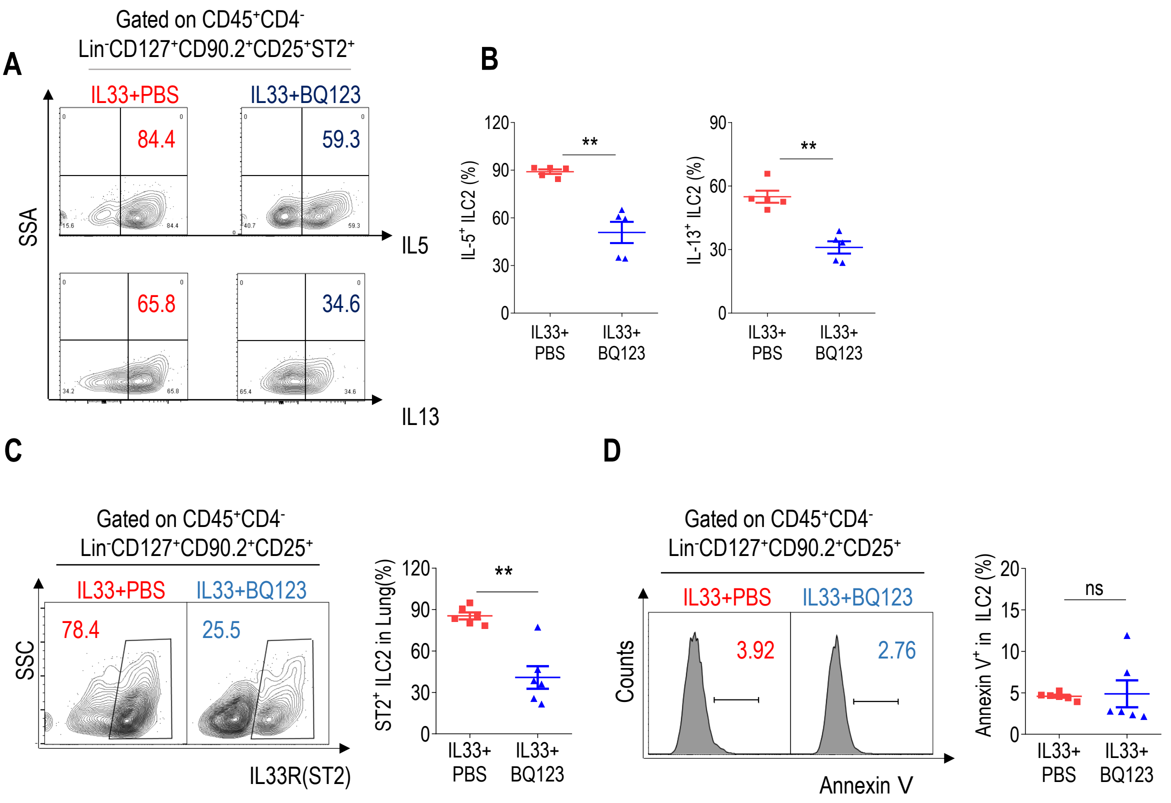

Supplementary Figure 5. ST2⁺ group 2 innate lymphoid cell (ILC2) populations changed following IL33-induced allergic lung inflammation.

(A and B) Representative flow cytometry plots (A) and statistical analysis (B) of intracellular IL-5 and IL-13 positive population in ILC2s. (C and D) Representative flow cytometry results (left) and statistical analysis (right) for ST2⁺ ILC2 (C) and apoptotic (D) cells are shown. Note: ns, not significant; *P < 0.05; **P < 0.01; ***P < 0.001; ****P < 0.0001. In all panels, individual results and mean ± standard error of the mean (SEM) are shown; statistical significance was determined using a two-tailed unpaired Student's t test (B) or Mann-Whitney test (B, C, and D).

Supplementary Figure 6:

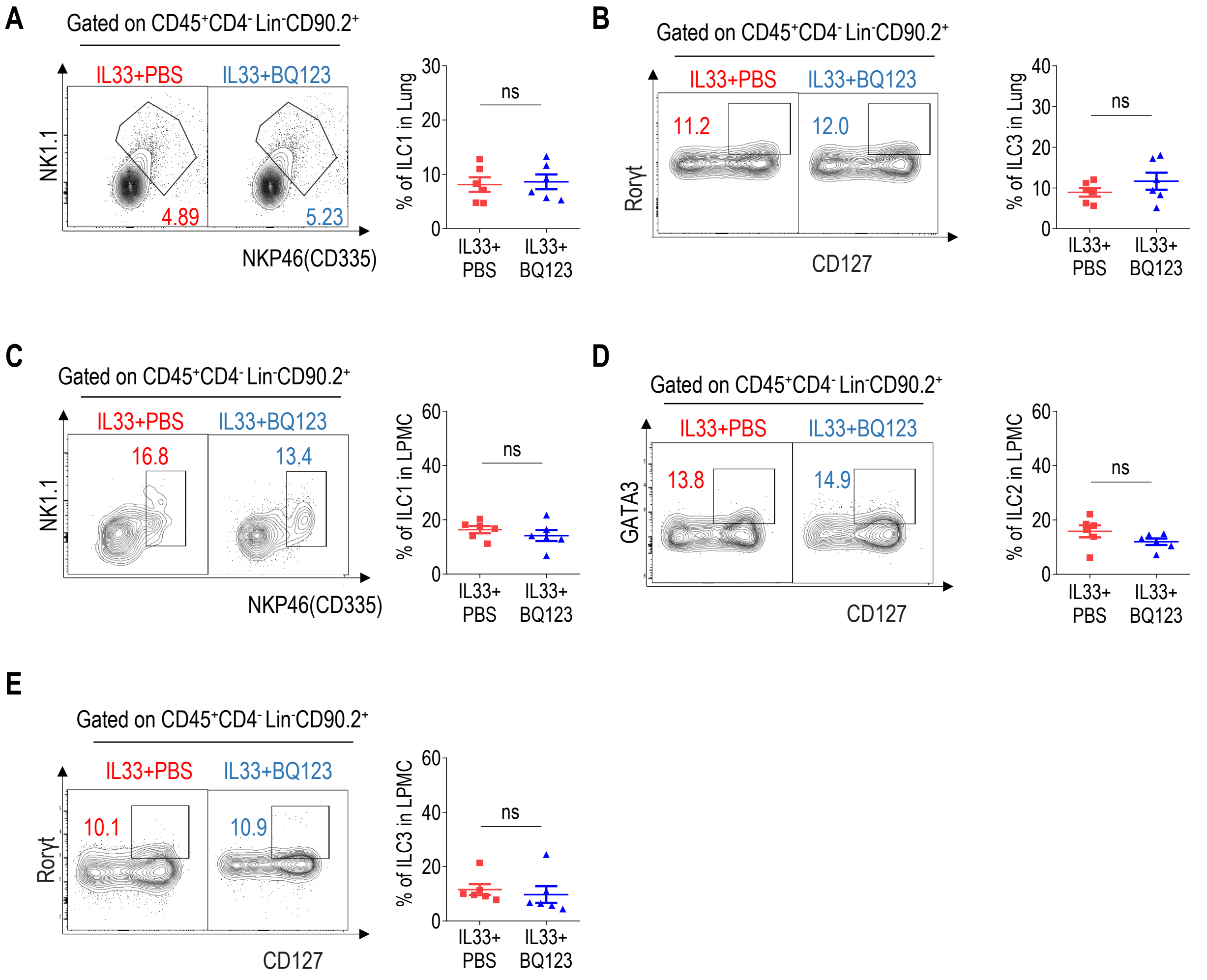

Supplementary Figure 6. BQ123 did not affect the changes related to IL33-induced allergic lung inflammation in other subgroups of innate lymphoid cells (ILCs).

(A and C) Representative flow cytometry results (left) and statistical analysis (right) of lung and LI ILC1s cells ($CD45^+$ $CD4^-$ Lin^- $CD90.2^+$ $NK1.1^+$ $NKP46^+$) are shown. (B and E) Representative flow cytometry results (left) and statistical analysis (right) of lung and LI ILC3s ($CD45^+$ $CD4^-$ Lin^- $CD90.2^+$ $CD127^+$ $Ror\gamma t^+$) are shown. (D) Representative flow cytometry results (left) and statistical analysis (right) of LI ILC2s ($CD45^+$ $CD4^-$ Lin^- $CD90.2^+$ $CD127^+$ $GATA3^+$) are shown. Data are representative of two independent experiments (n = 6 for IL33 + PBS group; n = 6 for IL33 + BQ123 group). ns, not significant; *P < 0.05; **P < 0.01; ***P < 0.001; ****P < 0.0001. In all panels, individual results and mean ± standard error of the mean (SEM) are shown; statistical significance was determined using a two-tailed unpaired Student's t test (A, B, C, D, and E).

Supplementary Figure 7:

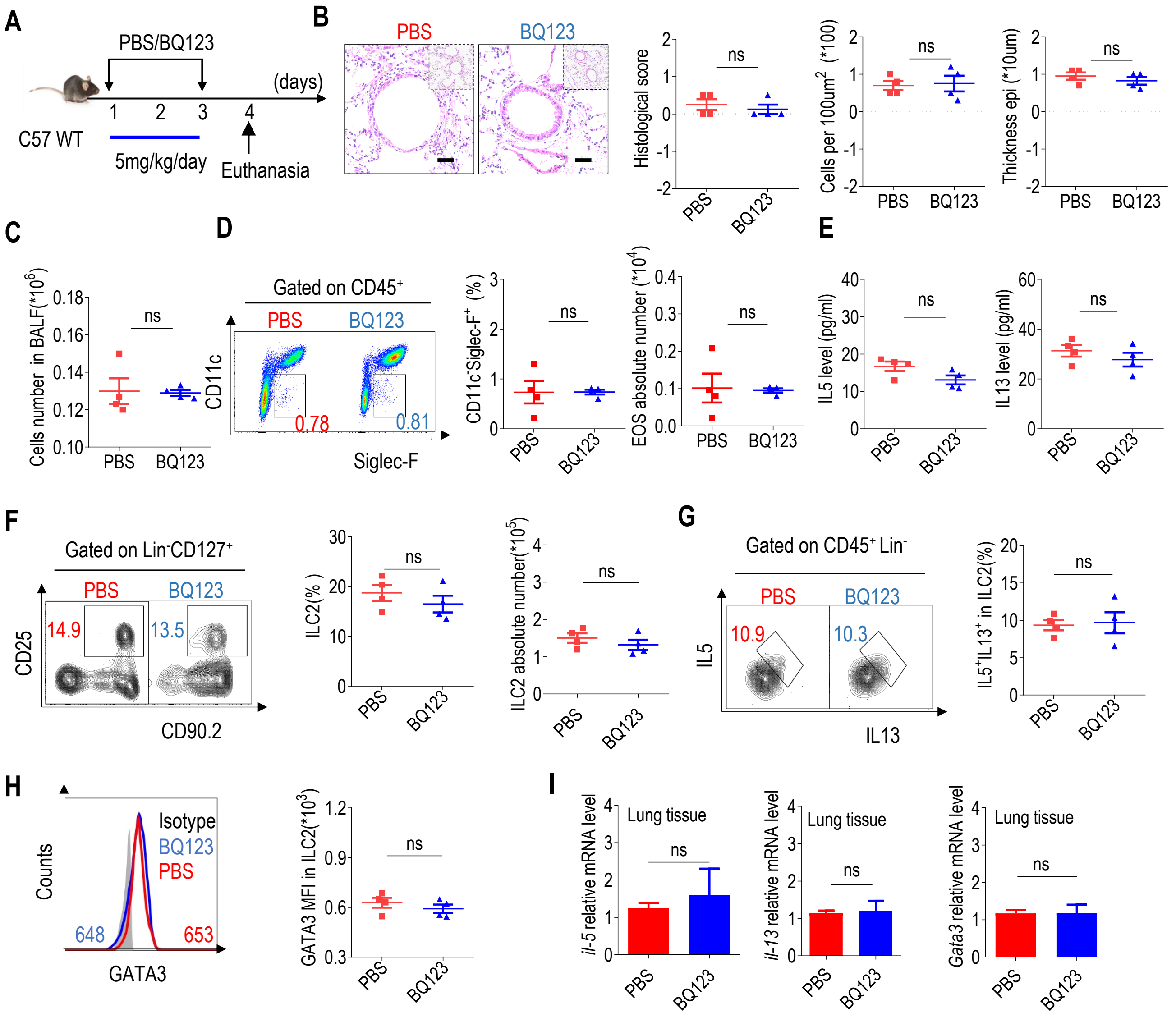

Supplementary Figure 7. BQ123 had no effect on group 2 innate lymphoid cells (ILC2s) under physiological conditions.

(A) A cohort of C57BL/6J mice was intranasally challenged with phosphate-buffered saline (PBS) and treated with BQ123 or PBS control for three days, as shown in the timeline. (B) Representative hematoxylin and eosin (H&E) staining of lung sections and inflammation scores (n = 4), including the infiltrating cells and airway epithelium thickness. Bars, 100μm. (C) Absolute number of BALF. (D) Typical example of flow cytometry and statistical results both population and the absolute number of EOS in the BALF. (E) Levels of IL-5 and IL-13 in BALF (n = 4). (F-H) Representative results of flow cytometry (left) and statistical analysis (right) of the frequencies of ILC2s (F), including absolute counts (F), $IL\text{-}5^{+}$ $IL\text{-}13^{+}$ ILC2s (G), and levels of GATA3 protein (H) in the lungs (n = 4). (I) mRNA expression levels of *Il5*, *Il13*, and *Gata3* were evaluated (n = 3). Note：ns, not significant; *P < 0.05; **P < 0.01; ***P < 0.001; ****P < 0.0001. In all panels, individual results and mean ± standard error of the mean (SEM) are shown; statistical significance was determined using a two-tailed unpaired Student’s t test (B, C, D, E, F, G, H, and I).

Supplementary Figure 8:

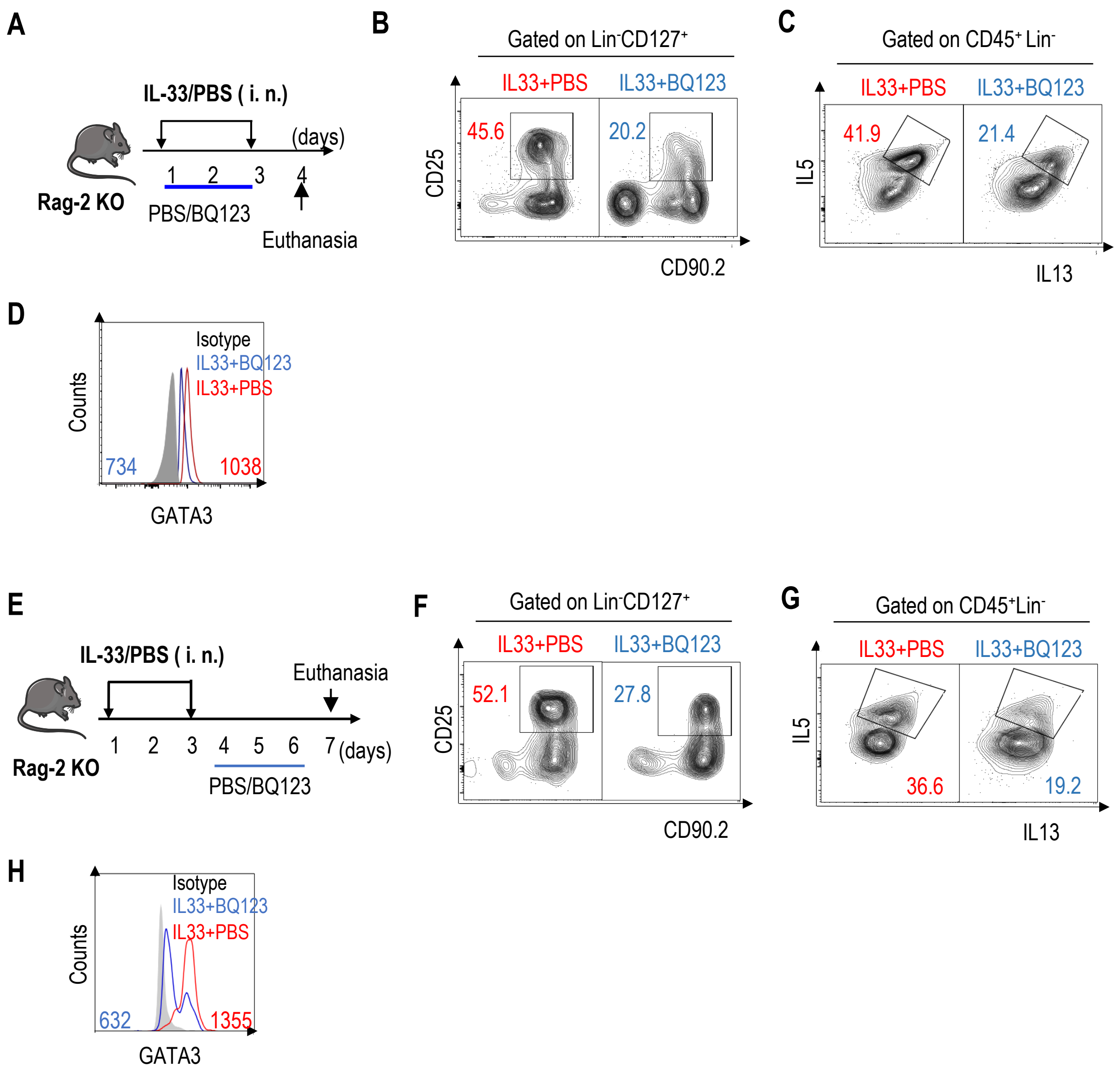

Supplementary Figure 8. BQ123 abrogated allergic lung inflammation in a T-cell-independent manner. (A and E) Experimental scheme. (B, C, F, G) Representative flow cytometry results of the frequencies of ILC2s (B and F) and IL-5^{+} IL-13^{+} ILC2s (C and G). (D and H) Typical example of flow cytometry of GATA3 protein levels in lung ILC2s.

Supplementary Figure 9:

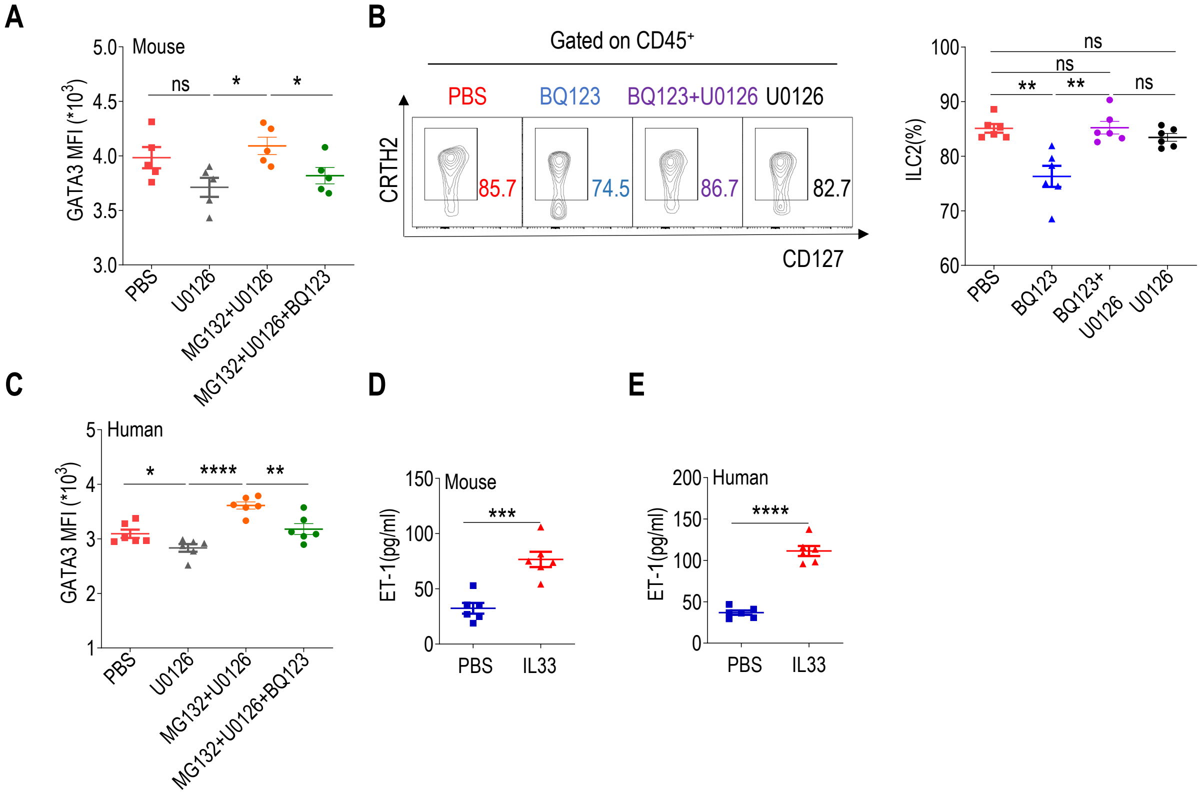

Supplementary Figure 9. The GATA3 stabilization was impaired in BQ123-mediated inhibition of the effector function of ILC2s. (A) Naive pulmonary ILC2s from C57BL/6J mice were sorted and subsequently co-cultured with or without the U0126/MG132/BQ123 in the presence of recombinant rmIL-2, rmIL-7, and rmIL-33 antibodies for 72 h respectively. Typical statistical results of intranuclear protein expression levels of transcription factor GATA3. (B) Representative flow cytometry results (left) and statistical analysis (right) of the population of human ILC2s are shown. (C) Human peripheral blood ILC2s were sorted and subsequently co-cultured with or without the U0126/MG132/BQ123 in the presence of recombinant rhIL-2, rhIL-7, and rhIL-33 antibodies for 72 h respectively. Typical statistical results of intranuclear protein expression levels of transcription factor GATA3. (D and E) The content of endothelin-1 (ET-1) in culture supernatant were evaluated (n = 6). Note: ns, not significant; *P < 0.05; **P < 0.01; ***P < 0.001; ****P < 0.0001. In all panels, individual results and mean ± standard error of the mean (SEM) are shown. Statistical significance was determined using a two-tailed unpaired Student's t test (A, B ,C, D and E).

Supplementary Figure 10:

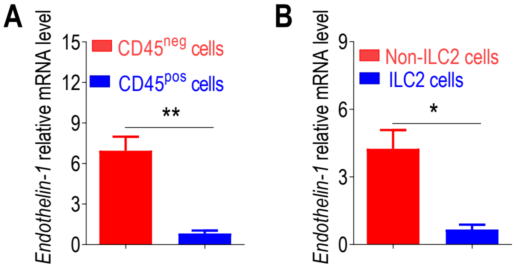

Supplementary Figure 10. ET-1 is highly expressed in both non-immune and non-group 2 innate lymphoid cells (ILC2s).

(A, B) mRNA expression levels of *Endothelin-1* were determined; *β-actin* level was used for normalization, and the lowest expression level was set to 1 (n = 3). Note: ns, not significant; *P < 0.05; **P < 0.01; ***P < 0.001; ****P < 0.0001. In all panels, individual results and mean ± standard error of the mean (SEM) are shown. Statistical significance was determined using a two-tailed unpaired Student's t test (A, and B).

Supplementary Figure 11:

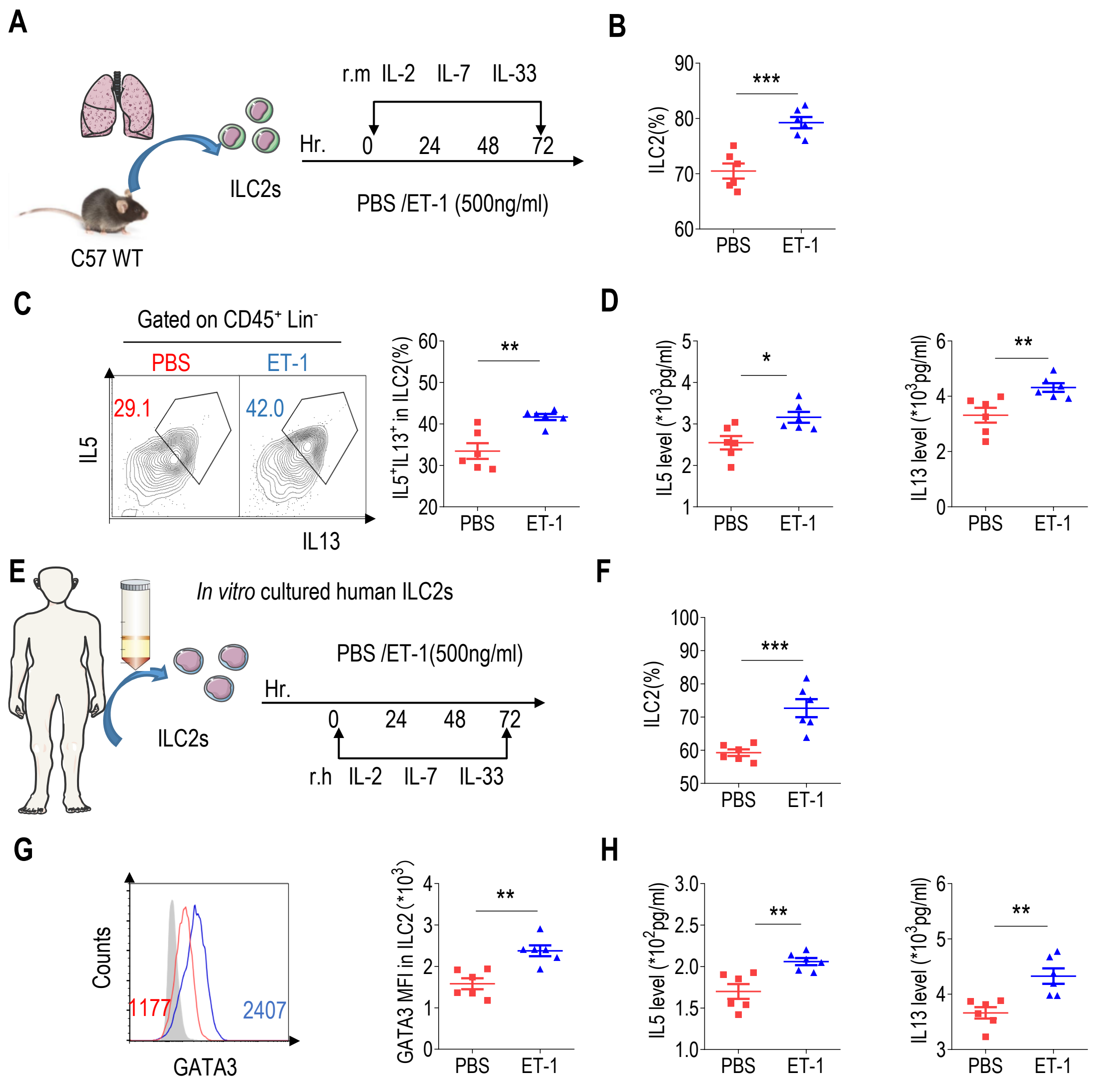

Supplementary Figure 11. ET-1 enhanced the effector function of group 2 innate lymphoid cells (ILC2s) i*n vitro*. (A) ILC2s were sorted from C57BL/6J mice and subsequently co-cultured with ET-1 (500 ng/ml) in the presence of rmIL-2, rmIL-7, and rmIL-33 for 72 h. (B and C) Representative statistical results for frequencies of ILC2s (B) and IL-5^+ IL-13^+ ILC2s (C) are shown (n = 6). (D) The levels of IL-5 and IL-13 in the co-culture supernatants (n = 6). (E) ILC2s sorted from human peripheral-blood ILC2s and induced *in vitro* in the presence of 20 ng of recombinant human (rh) IL-2, rhIL-7, and rhIL-33 for 72 h with or without ET-1 (n = 6). (F and G) Representative statistical analysis for the proportion (F) and GATA3 protein levels (G) (n = 6). (H) The levels of IL-5 and IL-13 in the co-culture supernatants (n = 6). Note: ns, not significant; * $P < 0.05$; ** $P < 0.01$; *** $P < 0.001$; **** $P < 0.0001$. In all the panels, individual results and mean ± standard error of the mean (SEM) are shown; statistical significance was determined using a two-tailed unpaired Student's t test (B, C, D, F, G, and H).
